# The SET7 protein of *Leishmania donovani* moderates the parasite’s response to a hostile oxidative environment

**DOI:** 10.1016/j.jbc.2024.105720

**Published:** 2024-02-02

**Authors:** Jyoti Pal, Varshni Sharma, Arushi Khanna, Swati Saha

**Affiliations:** Department of Microbiology, University of Delhi South Campus, New Delhi, India

**Keywords:** *Leishmania donovani*, trypanosome, SET domain, SET proteins, SET7, oxidative stress, protozoan parasite

## Abstract

SET domain proteins methylate specific lysines on proteins, triggering stimulation or repression of downstream processes. Twenty-nine SET domain proteins have been identified in *Leishmania donovani* through sequence annotations. This study initiates the first investigation into these proteins. We find LdSET7 is predominantly cytosolic. Although not essential, *set7* deletion slows down promastigote growth and hypersensitizes the parasite to hydroxyurea-induced G1/S arrest. Intriguingly, *set7*-nulls survive more proficiently than *set7*^+/+^ parasites within host macrophages, suggesting that LdSET7 moderates parasite response to the inhospitable intracellular environment. *set7*-null *in vitro* promastigote cultures are highly tolerant to hydrogen peroxide (H_2_O_2_)-induced stress, reflected in their growth pattern, and no detectable DNA damage at H_2_O_2_ concentrations tested. This is linked to reactive oxygen species levels remaining virtually unperturbed in *set7*-nulls in response to H_2_O_2_ exposure, contrasting to increased reactive oxygen species in *set7*^+/+^ cells under similar conditions. In analyzing the cell’s ability to scavenge hydroperoxides, we find peroxidase activity is not upregulated in response to H_2_O_2_ exposure in *set7*-nulls. Rather, constitutive basal levels of peroxidase activity are significantly higher in these cells, implicating this to be a factor contributing to the parasite’s high tolerance to H_2_O_2_. Higher levels of peroxidase activity in *set7*-nulls are coupled to upregulation of tryparedoxin peroxidase transcripts. Rescue experiments using an LdSET7 mutant suggest that LdSET7 methylation activity is critical to the modulation of the cell’s response to oxidative environment. Thus, LdSET7 tunes the parasite’s behavior within host cells, enabling the establishment and persistence of infection without eradicating the host cell population it needs for survival.

The SET domain proteins get their name from the three *Drosophila* proteins the domain was first identified in: suppressor of variegation [Su(var)3-9], enhancer of zeste [E(z)], trithorax (reviewed in (1)). Initially identified as proteins that methylate histones at specific lysine residues, SET proteins were subsequently found to target non-histone substrates as well, and can mediate monomethylation, dimethylation, and/or trimethylation of their target residues. Common histone targets include H3K4, H3K9, H3K27, H3K36, and H4K20 (reviewed in (1, 2, 3)). The impacts of these histone methylation events vary widely, and thus while H3K4 methylation is associated with transcriptional upregulation, H3K9 and H3K27 methylation marks mediate transcriptional repression. Non-histone substrates include tumor suppressors (such as p53 and Rb), transcription factors (like GATA4 and TAF_10_), and signaling proteins (such as STAT3) (reviewed in (1, 2, 3)). SET domain proteins modulate a wide range of cellular processes, such as DNA replication, DNA repair, transcriptional activation as well as silencing, mRNA splicing, heterochromatin formation, and X-chromosome inactivation. Abrogation of SET domain protein functions have been linked to various types of cancer (1, 3).

While exhaustively studied in yeast and mammalian cells, limited information is available about protozoan SET proteins. The most widely investigated are the *Plasmodium falciparum* SET proteins, where six of the ten proteins identified appear to be essential for survival of blood stage parasites. The histone target residues of six PfSET proteins have been identified and their functional roles characterized to varying degrees (4, 5, 6). The *Tetrahymena* EZL3 SET protein has been found to modulate development and progeny viability (7). The non-nuclear *Toxoplasma* SET domain protein modulates host cell invasion as well as the parasite’s exit from the host cell (8). *In vitro* biochemical assays have uncovered the target residues of three SET domain proteins identified in *Entamoeba histolytica*, two of which appear to be involved in phagocytosis (9).

*Leishmania* species are trypanosomatids that are endemic to 88 countries and according to WHO around 12 million people are at risk globally (https://www.who.int/news-room/fact-sheets/detail/leishmaniasis). Leishmaniases are manifested in three forms: cutaneous, subcutaneous, and visceral and different *Leishmania* species cause the different forms of the disease. In South Asia the prevalent form of the disease is visceral leishmaniasis or kala-azar, caused by *Leishmania donovani.* Visceral leishmaniasis is treatable, but treatment regimens are lengthy, expensive, and have toxic side effects. Adding to that the problems of growing drug resistance, risks due to HIV-*Leishmania* coinfection, and emerging threat of post kala-azar dermal leishmaniasis in the Indian subcontinent, this parasite’s cellular biology remains an area of interest to several research groups around the world. While no one has directly investigated the role of SET domain proteins in *Leishmania* species, the *Leishmania tarentolae* SET domain protein LtaP35.2400 has been found to be a part of a multiprotein complex that also carries the JBP3 protein, which has been previously shown to play a role in regulating transcription termination events (10). Twenty nine SET domain proteins have been identified in *Trypanosoma brucei* by whole-genome sequence annotations (11), and through orthology searches in the Tritryp DB (www.tritrypdb.org) we have been able to identify orthologs of all 29 in *L. donovani*. The subcellular localization of the *T. brucei* SET proteins has been examined in blood form as well as procyclic form (PF), by epitope-tagging coupled to immunofluorescence (11, 12, 13). Eight of the twenty-nine proteins localize to the nucleus in the blood form parasites, though also found in the cytoplasm. Four of these eight proteins localize to the nucleus in the PF parasites as well. Six SET proteins localize to the nucleus (though also found in the cytoplasm) in the PF parasites only. Two proteins, TbSET26 and TbSET27, have been found to be enriched at the transcriptional start regions (TSRs) (11). Furthermore, TbSET27 has been found to exist as part of a multiprotein complex (SPARC) that modulates the accuracy of transcription initiation at the TSRs (14).

This report presents the data from the first study across *Leishmania* species that directly investigates a SET protein, examining the functional role of the protein encoded by the *L. donovani* 1S ortholog of the gene LdBPK_360230.1, which we name LdSET7 as it is the *Leishmania* ortholog of the TbSET7 protein. Our study reveals that the gene encoding LdSET7 (*set7*) is not essential for cell survival. The LdSET7 protein is found to play a role in mediating the parasite’s response to an oxidative environment in both promastigotes (the extracellular form in insect host) and amastigotes (the intracellular form in mammalian host).

## Results

### *Leishmania donovani* SET7 is constitutively expressed and is predominantly cytosolic

In the absence of genome sequence information of *L. donovani* 1S (Ld1S), the *set7* gene (∼1.42 kb) of Ld1S was cloned by amplification using genomic DNA as template with primers designed against its ortholog in *L. donovani* BPK282A1 (LdBPK_360230.1), whose sequence was obtained from TriTrypDB ((15), www.tritrypdb.org). The cloned amplicon was sequenced to confirm its veracity (GenBank Accession no: OR479702). It was found to carry only one SNP in comparison with the LdBPK_360230.1 gene, which resulted in a change from leucine to valine. A comparison of the derived amino acid sequence of LdSET7 with orthologs of the same protein in other trypanosomatid species using Clustal Omega analysis (16) and blastp (https://blast.ncbi.nlm.nih.gov.in) revealed that LdSET7 showed 35 to 40% identity over a coverage of 91% with SET7 of *Trypanosoma* species and 90 to 99% identity with SET7 of other *Leishmania* species over 100% coverage (Fig. S1). Analysis of the SET7 amino acid sequence revealed that the ∼51.5 kDa protein carries a SET domain between amino acids 304 to 422, and a post-SET domain at the C-terminal end between residues 430 to 446 (Fig. S2*A*). SET domains are often flanked by pre-SET and post-SET domains. While the pre-SET domain is believed to overall help stabilize the SET protein structure *via* its interactions with the residues of the SET domain, the post-SET domain forms part of the active site hydrophobic channel (17).

The digenetic *Leishmania* parasites exist extracellularly as promastigotes in the insect host, initially as free-living flagellate parasites in the midgut and subsequently in the salivary glands of the insect, whereupon they are introduced into the mammalian host when the insect feeds on the host’s blood. In the mammalian host the parasite is taken up by macrophages where they take up residence and propagate as intracellular aflagellate amastigotes. The expression of LdSET7 in logarithmically growing as well as stationary phase *L. donovani* promastigotes was analyzed in whole-cell extracts using anti-LdSET7 antibodies already available in the lab and found to be comparable at both stages (Fig. S2*B*). Examination of whole-cell lysates isolated from procyclics (early stage promastigotes normally existing in insect midgut) and metacyclics (late stage promastigotes found usually in insect salivary glands; the infective stage) revealed that expression was more or less equivalent in both types of promastigotes (Fig. S2*C*). Upon examining LdSET7 expression at different stages of the cell cycle using promastigotes that had been synchronized at the G1/S boundary with a hydroxyurea (HU) block and then released into S phase (described in Experimental procedures), it was observed that LdSET7 expression was maximum in S phase (Fig. S2*D*). This was in contrast to expression of another *L. donovani* SET protein, encoded by LdBPK_212120.1 (ortholog of TbSET29 and thus named LdSET29), which was expressed more or less equivalently at all stages of cell cycle (Fig. S2*D*). As the antibodies did not work in immunocytochemistry experiments, the *set7* gene was expressed in fusion with a C-terminal FLAG tag in *L. donovani* promastigotes (Fig. S3*A*), and the subcellular localization of LdSET7-FLAG analyzed by indirect immunofluorescence as described in Experimental procedures, using kinetoplast morphology and segregation pattern as cell cycle stage marker (18). LdSET7-FLAG was found to be primarily cytosolic at all stages of the cell cycle (Fig. S3*B*). Collectively, these data demonstrate that LdSET7 is robustly expressed in promastigotes (in both procyclics and metacyclics) and is constitutively expressed, predominantly in the cytoplasm, with maximal expression detectable in S phase.

### Parasites partially depleted of LdSET7 display greater competency in survival within host macrophages than WT parasites

**Figure 1:**
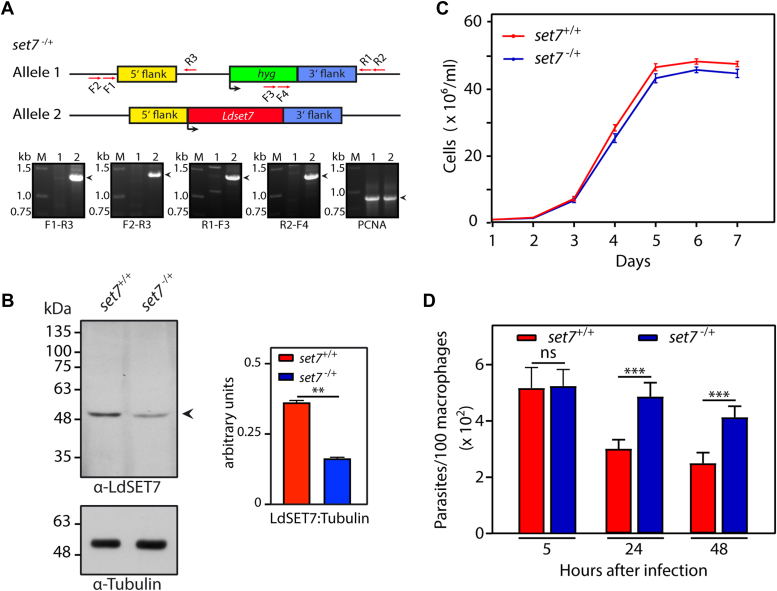
**Effect of partial depletion of LdSET7 on parasite growth and survival.***A*, creation of *set7*^*−/+*^. Recombination at both ends was checked by PCRs across the deletion junctions. Positions of primers used are indicated with *arrows*. F1, F2, F3, F4: forward primers. R1, R2, R3: reverse primers. PCNA served as input template DNA control. *Lanes 1*: Ld1S genomic DNA template. *Lanes 2*: *set7*^*−/+*^ genomic DNA template. M: DNA ladder. *B*, Western blot analysis of whole-cell extracts isolated from 1 × 10^8^ logarithmically growing promastigotes using anti-SET7 antibodies (1:2500 dilution). Loading control: tubulin. Quantitation was carried out using ImageJ (https://imagej.net/ij/) analysis and plotted values represent average of two experiments. Error bars represent SD, and statistical significance was determined using two-tailed unpaired student’s *t* test. ∗∗ represents *p* value <0.005. *C*, analysis of growth of parasites. Values plotted represent average of three experiments. Each experiment was performed with two technical replicates. Error bars represent SD. *D*, analysis of parasite survival in host macrophages. Z-stack imaging using confocal microscopy was employed to count the number of intracellular parasites (4′,6-diamidino-2-phenylindole-stained nuclei served as marker). The experiment was performed thrice and average values are plotted in the bar graph. Error bars depict SD, and two-tailed unpaired student’s *t* test was used to assess statistical significance. ∗∗∗*p* value <0.0005. ns: statistically not significant. *set7*^*+/+*^ cells: Ld1S::hyg cells. *set7*^*−/*+^ cells: *set7-*heterozygous KO cells. Ld1S, *Leishmania donovani* 1S.

To investigate the role of LdSET7 in the *Leishmania* cell, we replaced the *set7* genomic alleles sequentially by homologous recombination (as detailed in Experimental procedures). The first allele was replaced with a hygromycin resistance cassette and the authenticity of recombination at both ends verified by PCRs across the deletion junctions (Fig. 1*A*). Western blot analysis revealed a corresponding decrease in the levels of the LdSET7 protein (Fig. 1*B*). Analysis of the growth pattern of these *set7-*heterozygous knockout (*set7*^−/+^) promastigotes revealed that the observed reduction in LdSET7 expression upon knockout of one *set7* allele did not have any impact on growth and survival under normal conditions (Fig. 1*C*). The cell cycle progression pattern of *set7*^−/+^ cells, synchronized at the G1/S boundary with HU, and then released into S phase (as described in Experimental procedures), was similar to that of *set7*^+/+^ cells (Fig. S4). To examine the effect of loss of one *set7* allele on amastigote growth and propagation, we infected *set7*^−/+^ parasites into murine macrophage cells J774A.1 (as described in Experimental procedures) and followed their survival over 48 h after infection. As seen in Figure 1*D*, the intracellular load of *set7*^−/+^ parasites was comparable to that of *set7*^+/+^ parasites at the end of the 5 h parasite-host cell incubation period (5 h time point), signifying that the ability to infect host cells *per se* was not altered in *set7*^−/+^ cells. However, unexpectedly, *set7*^−/+^ parasites survived more competently than *set7*^+/+^ parasites within the host cells, as determined by the higher intracellular parasite load 24 h and 48 h after infection (Fig. 1*D*). Thus, the levels of LdSET7 protein in *set7*^*−/+*^ parasites were sufficient for the promastigotes to remain unperturbed under normal *in vitro* growth conditions but insufficient to maintain the normal parasite-host cell balance in case of the amastigotes.

### Promastigotes partially depleted of LdSET7 are more tolerant to an oxidative environment than WT promastigotes

**Figure 2:**
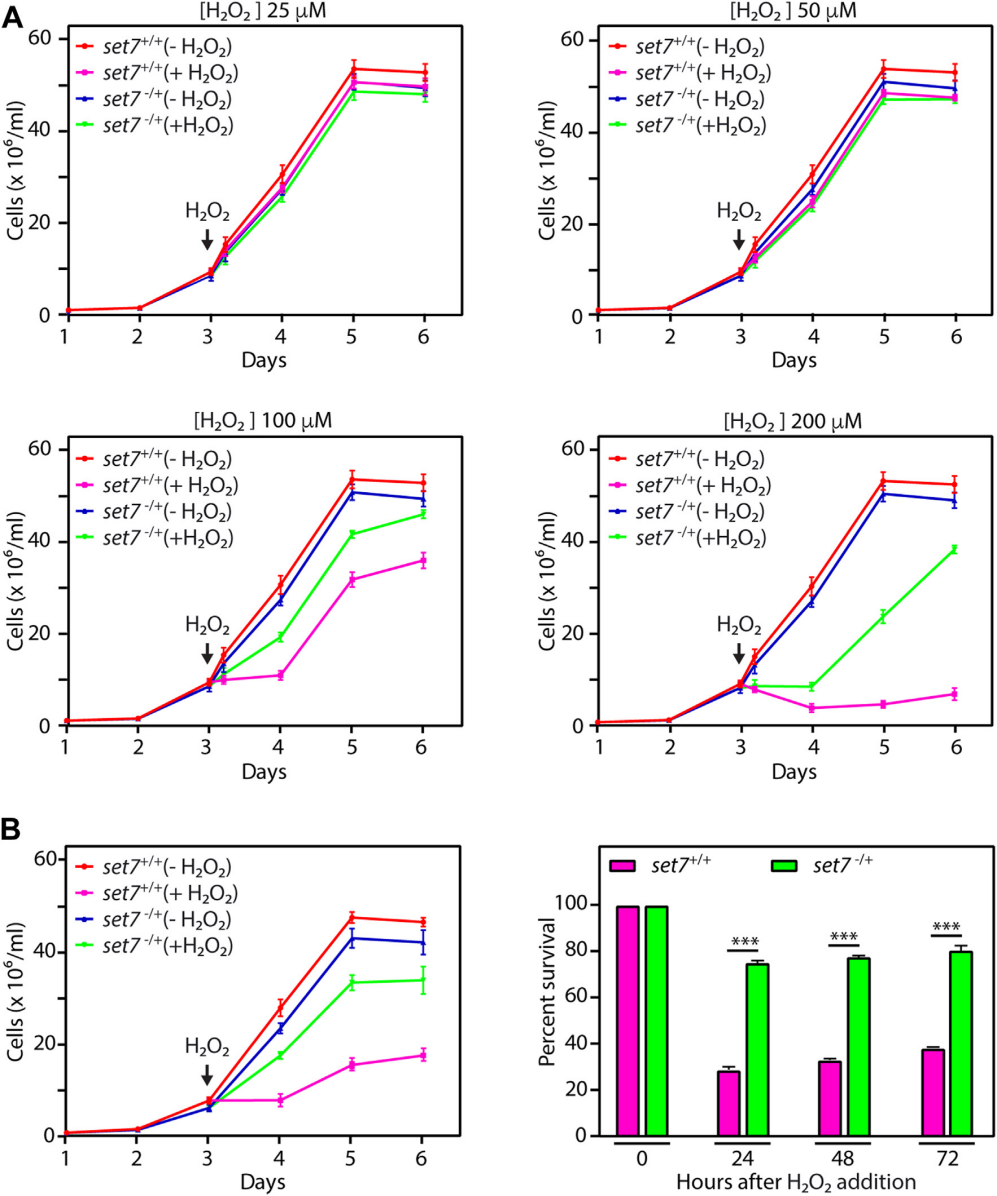
**Effect of LdSET7 depletion on promastigote growth and survival in response to H**_**2**_**O**_**2**_**exposure.***A*, cultures were initiated from stationary phase cultures, and 0 to 200 μM H_2_O_2_ was added to the cultures 48 h after initiation. At the time of addition of H_2_O_2_, the cultures were split into two, with half the cells receiving H_2_O_2_ treatment and the other half being carried forward as the untreated culture. After a 5-h exposure to H_2_O_2,_ the parasites were refed with fresh H_2_O_2_-free medium, and cells were counted every 24 h. *Graphs* show growth patterns of the cells. The experiment was done thrice, with two technical replicates in each experiment. Values plotted are average of three experiments, and error bars depict SD. The experimental data has been split into four panels for easier viewing and thus the (-H_2_O_2_) data is identical in all four panels. *B*, cultures were initiated from stationary phase cultures, and 100 μM H_2_O_2_ was added to the cultures 48 h after initiation. At the time of addition of H_2_O_2_, the cultures were split into two, with half the cells receiving H_2_O_2_ treatment and the other half being carried forward as the untreated culture. H_2_O_2_-treated cultures were maintained in H_2_O_2_-containing medium, with H_2_O_2_ being replenished every 10 h, and cells were counted every 24 h. *Left panel*: growth analysis of the cultures. *Right panel*: Survival percent of H_2_O_2_-treated cultures with reference to untreated cultures was determined by dividing the number of cells in treated cultures by the number of cells in untreated cultures and multiplying by 100. The experiment was done thrice, with two technical replicates in each experiment. Values plotted are average of three experiments, and error bars depict SD. Student’s *t* test (two-tailed unpaired) was used to assess statistical significance. ∗∗∗*p* value <0.0005. *set7*^*+/+*^ cells: Ld1S::hyg cells. *set7*^*−/*+^ cells: *set7-*heterozygous KO cells. H_2_O_2_, hydrogen peroxide; Ld1S, *Leishmania donovani* 1S.

Considering the oxidative intracellular environment of macrophage host cells, we examined the growth of *set7*^−/+^ promastigotes in conditions where oxidative stress has been induced. Thus, *set7*^−/+^ parasite cultures were initiated from stationary phase cultures, and hydrogen peroxide (H_2_O_2_) varying over 25 to 200 μM was added on day 3 (as described in Experimental procedures). The parasites were incubated in this H_2_O_2_-containing medium (M199) for 5 h before replacing it with fresh H_2_O_2_-free medium and continuing incubation. Growth was monitored over the next few days, and on comparing the behavior of *set7*^−/+^ promastigotes with that of *set7*^+/+^ promastigotes, it was observed that while neither cell type displayed any obvious response to lower concentrations of H_2_O_2_ (25–50 μM), they behaved quite differently in response to higher H_2_O_2_ concentrations. *set7*^+/+^ promastigotes grew slower in response to 100 μM H_2_O_2_ treatment and displayed severely compromised growth in response to 200 μM H_2_O_2_ treatment; in contrast, *set7*^−/+^ parasites displayed a very moderate response to 100 μM H_2_O_2_ treatment, and were able to recover and continue to grow well in response to 200 μM H_2_O_2_ treatment as well (Fig. 2*A*). The altered behavior of LdSET7-depleted parasites upon exposure to H_2_O_2_ was more apparent when parasites were incubated in 100 μM H_2_O_2_ over several days (with replenishment of H_2_O_2_ every 10 h), wherein growth of *set7*^+/+^ cells was distinctly compromised while *set7*^−/+^ cells continued to thrive (Fig. 2*B*). These data indicate that LdSET7 regulates the parasite’s response to an oxidative milieu under *in vitro* conditions as well.

### The set7 gene is not essential for cell survival

**Figure 3:**
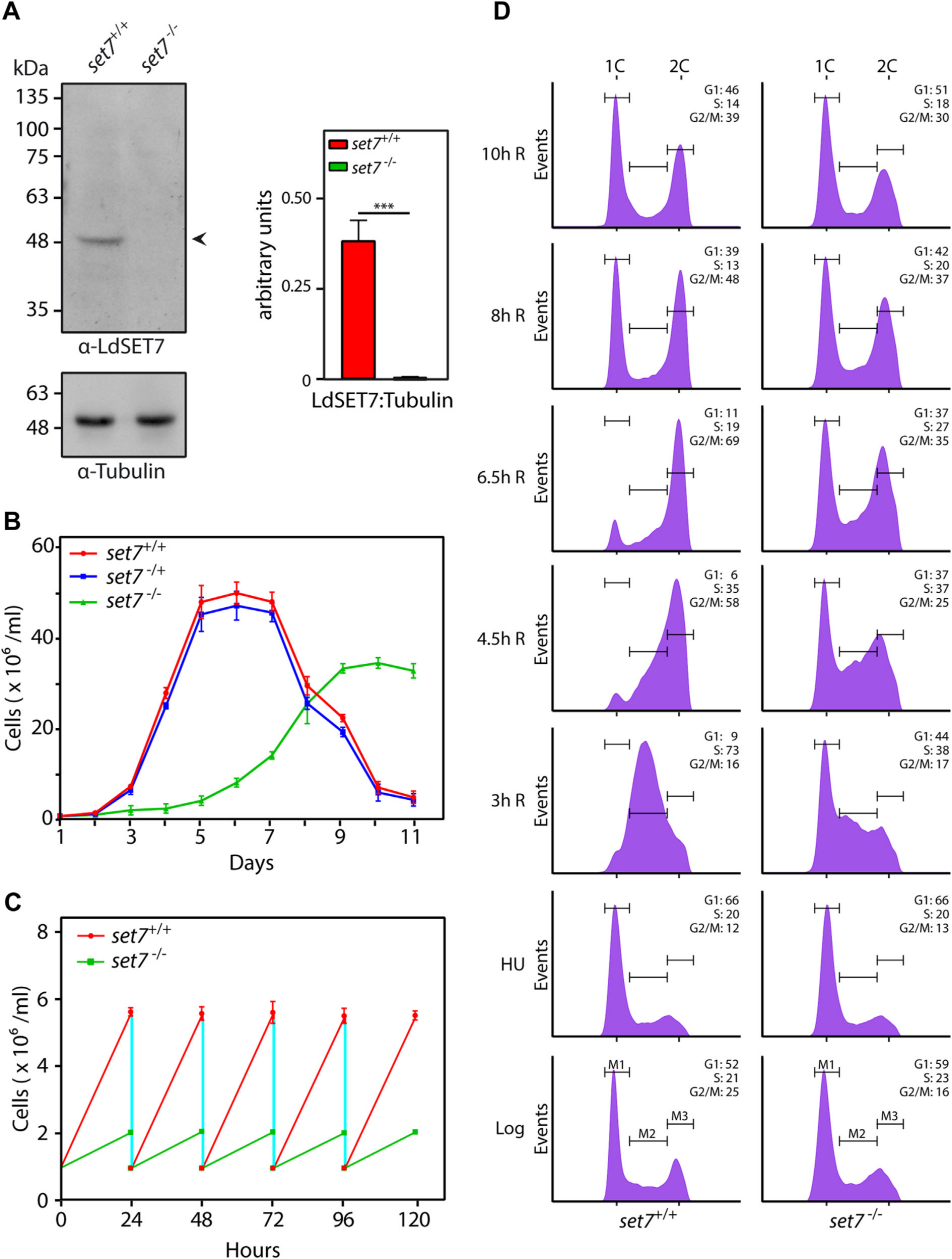
**Effect of elimination of *set7* gene on promastigote growth and cell cycle progression.***A*, Western blot analysis of whole-cell extracts isolated from 1 × 10^8^ logarithmically growing promastigotes using anti-SET7 antibodies (1:2500 dilution). Loading control: tubulin. Quantitation was carried out using ImageJ analysis and plotted values represent average of three experiments. Error bars represent SD, and statistical significance was determined using student’s *t* test. ∗∗∗ represents *p* value <0.0005. *B*, analysis of growth of parasites. Cultures were initiated at 1 × 10^6^ cells/ml from stationary phase cultures. Values plotted represent average of three experiments. Each experiment was performed with two technical replicates. Error bars represent SD. *C*, generation time of cells was determined by initiating cultures from logarithmically growing cultures, at 1 × 10^6^ cells/ml, and diluting the cultures to 1 × 10^6^ cells/ml every 24 h after counting them. The experiment was done thrice and average values are plotted, with error bars depicting SD. *D*, flow cytometry analysis of HU-synchronized promastigotes. Time points at which cells were sampled are indicated on the *left* of each row of histograms. Thirty thousand events were analyzed at every time point. M1, M2, and M3 represent gating for cells in G1, S, and G2/M, respectively. Percent cells in each cell cycle phase are indicated in *upper right-hand corner* of each histogram. The experiment was done thrice, with comparable data, and data of one experiment are shown. *set7*^*+/+*^ cells: Ld1S::neo-hyg cells. *set7*^*−/*+^ cells: *set7-*heterozygous KO cells. *set7*^*−/−*^ cells: *set7*-nulls. HU, hydroxyurea; Ld1S, *Leishmania donovani* 1S.

The second *set7* allele was replaced with a *neo*^*r*^ cassette and genuine recombination at the 3′end checked by PCRs across the deletion junction, while the authenticity of recombination at the 5′end was checked by inverse PCRs across the deletion junction (Fig. S5). Western blot analysis confirmed the absence of LdSET7 expression in *set7*-nulls (*set7*^−/−^, Fig. 3*A*), and analysis of growth revealed that promastigotes grew slower when completely devoid of LdSET7, entering log phase 3 days later than *set7*^+/+^ cells and never reaching the same cell density as *set7*^+/+^ cells before entering stationary phase (Fig. 3*B*). The generation time of *set7*^−/−^ cells was found to be considerably longer than that of *set7*^+/+^ cells (∼23 h as compared to the usual ∼9.7 h; Fig. 3*C*). Examination of cell cycle progression patterns of HU-synchronized promastigotes by flow cytometry analysis, revealed that *set7*^−/−^ cells displayed a heightened sensitivity to HU-induced G1/S arrest, with a large fraction of the cells failing to be released into S phase upon removal of HU (Fig. 3*D*). While the fraction of cells that got released from the HU-induced block appeared to traverse S phase and G2/M in a manner comparable to control cells, no definitive conclusion regarding the cause for increased generation time could be drawn from this data. It is possible that the span of G1 phase is longer in *set7*^*−/−*^ cells; this needs further investigation. From the data in Figures 1 and 3, we concluded that under normal *in vitro* growth conditions while a partial depletion of LdSET7 (to ∼50% WT levels) did not have any impact on growth and cell cycle progression, complete elimination of LdSET7 expression slowed down growth and increased the generation time more than 2-fold. The survival of the parasite in the absence of the *set7* gene indicates that *set7* is not essential to the parasite.

### *set7*-nulls survive more proficiently than WT cells within host macrophages

**Figure 4:**
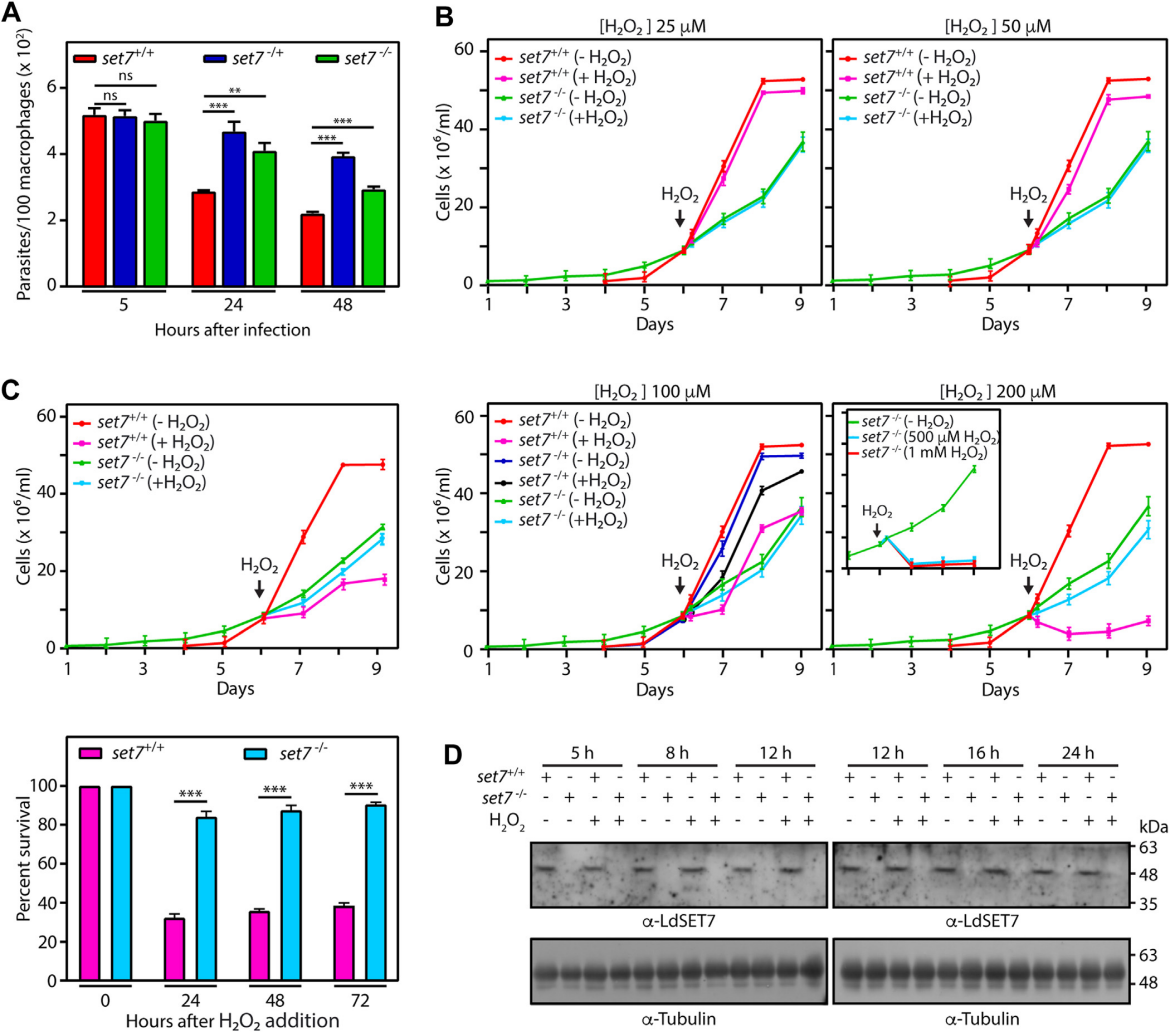
**Effect of oxidizing environment on *set7*-null parasites.***A*, analysis of parasite survival in host macrophages. Z-stack imaging using confocal microscopy was employed to count the number of intracellular parasites (4′,6-diamidino-2-phenylindole-stained nuclei served as marker). The experiment was performed thrice, and average values are plotted in the bar graph. Error bars depict SD, and student’s *t* test (two-tailed, unpaired) was used to assess statistical significance. ∗∗∗*p* value <0.0005, ∗∗*p* value <0.005, and ns: statistically not significant. *B*, effect of H_2_O_2_ exposure on promastigote growth and survival. Cultures were initiated from stationary phase cultures, and 0 to 200 μM H_2_O_2_ was added to the cultures at 7 to 9 × 10^6^ cells/ml. At the time of addition of H_2_O_2_ the cultures were split into two, with half the cells receiving H_2_O_2_ treatment and the other half being carried forward as the untreated culture. Parasites were re-fed with fresh H_2_O_2_ -free medium after a 5 h-exposure to H_2_O_2_, and cells counted every 24 h. The experiment was done thrice, with two technical replicates in each experiment. Values plotted are average of three experiments and error bars depict SD. The experimental data has been split into four panels for easier viewing and thus the (-H_2_O_2_) data is identical in all four panels. Inset in *lower right panel* shows effect of higher H_2_O_2_ concentrations on *set7*-null cultures. Cultures were initiated at 1 × 10^6^ cells/ml, and hydrogen peroxide (500 or 1000 μM) added on reaching a cell density of ∼7 to 9 × 10^6^ cells/ml (Day 6). Cells were counted every 24 h thereafter. *C*, effect of prolonged H_2_O_2_ exposure on promastigote growth and survival. Cultures were initiated from stationary phase cultures, 100 μM H_2_O_2_ added to the cultures at cell density 7 to 9 × 10^6^ cells/ml (at the time of addition of H_2_O_2_ the cultures were split into two, with half the cells receiving H_2_O_2_ treatment and the other half being carried forward as the untreated culture), and cultures maintained in H_2_O_2_-containing medium (with H_2_O_2_ being replenished every 10 h), with cells being counted every 24 h. *Upper panel*: growth analysis of the cultures. *Lower panel:* survival percent of H_2_O_2_-treated cultures with reference to untreated cultures, determined by dividing the number of cells in treated cultures by the number of cells in untreated cultures and multiplying by 100. The experiment was done thrice, with two technical replicates in each experiment. Values plotted are average of three experiments, and error bars depict SD. Student’s *t* test (two-tailed, unpaired) was used to assess statistical significance. ∗∗∗*p* value <0.0005. *D*, Western blot analysis of whole-cell extracts isolated from 8 × 10^7^ cells that were exposed to 100 μM H_2_O_2_ for 5 h, using anti-SET7 antibodies (1:2500 dilution). Loading control: tubulin. Time points indicate hours after start of the H_2_O_2_ exposure. *set7*^*+/+*^ cells: Ld1S::neo-hyg cells. *set7*^*−/*+^ cells: *set7-*heterozygous KO cells. *set7*^*−/−*^ cells: *set7*-nulls. H_2_O_2_, hydrogen peroxide; Ld1S, *Leishmania donovani* 1S.

Even more interestingly, these slower growing *set7*^−/−^ parasites (Fig. 3, *B* and *C*) survived more proficiently than *set7*^+/+^ parasites within host macrophages, though not to the same extent as *set7*^−/+^ parasites, reflecting the longer generation time of *set7*^−/−^ cells as compared to *set7*^−/+^ cells (Fig. 4*A*). Thus, LdSET7 appears to be moderating the parasite’s response to the inhospitable intracellular environment of host cells. The differential response of *set7*^−/−^ promastigotes to an oxidative growth environment *in vitro* was even more pronounced. While *set7*^−/−^ cells in general grew slower than *set7*^+/+^ promastigotes, the parasites did not demonstrate any apparent response to a 5-h H_2_O_2_ treatment over concentrations of 25 to 200 μM (Fig. 4*B*) and nor was any response evident when the parasites were incubated in 100 μM H_2_O_2_ over several days (Fig. 4*C*). When treated with H_2_O_2_ over concentrations of 500 to 1000 μM for 5 h, however, *set7*-nulls did not survive (Fig. 4*B*, inset in lower right panel). Elimination of LdSET7 from the cell thus appears to make the parasites extremely tolerant to the effects of an H_2_O_2_-induced oxidative environment.

The effect of H_2_O_2_ treatment on LdSET7 expression in *L. donovani* promastigotes was analyzed by incubating cells with H_2_O_2_ (100 μM) for 5 h, followed by continued growth in H_2_O_2-_free medium and isolation of whole-cell lysates at various time intervals thereafter. The lysates were analyzed using Western blotting with anti-SET7 antibodies, and as seen in Figure 4*D*, LdSET7 expression levels did not change in response to H_2_O_2_ treatment. To see if H_2_O_2_ treatment affected the subcellular localization of LdSET7, *L. donovani* promastigotes expressing LdSET7-FLAG were treated with H_2_O_2_ (100 μM) for 5 h, collected by centrifugation, and analyzed by indirect immunofluorescence. No change in subcellular localization was apparent (Fig. S6). The results presented in Figure 4 reinforce the fact that LdSET7 regulates the cell’s response to an oxidative milieu.

### LdSET7-depleted promastigotes do not display detectable DNA damage in response to an oxidative environment

**Figure 5:**
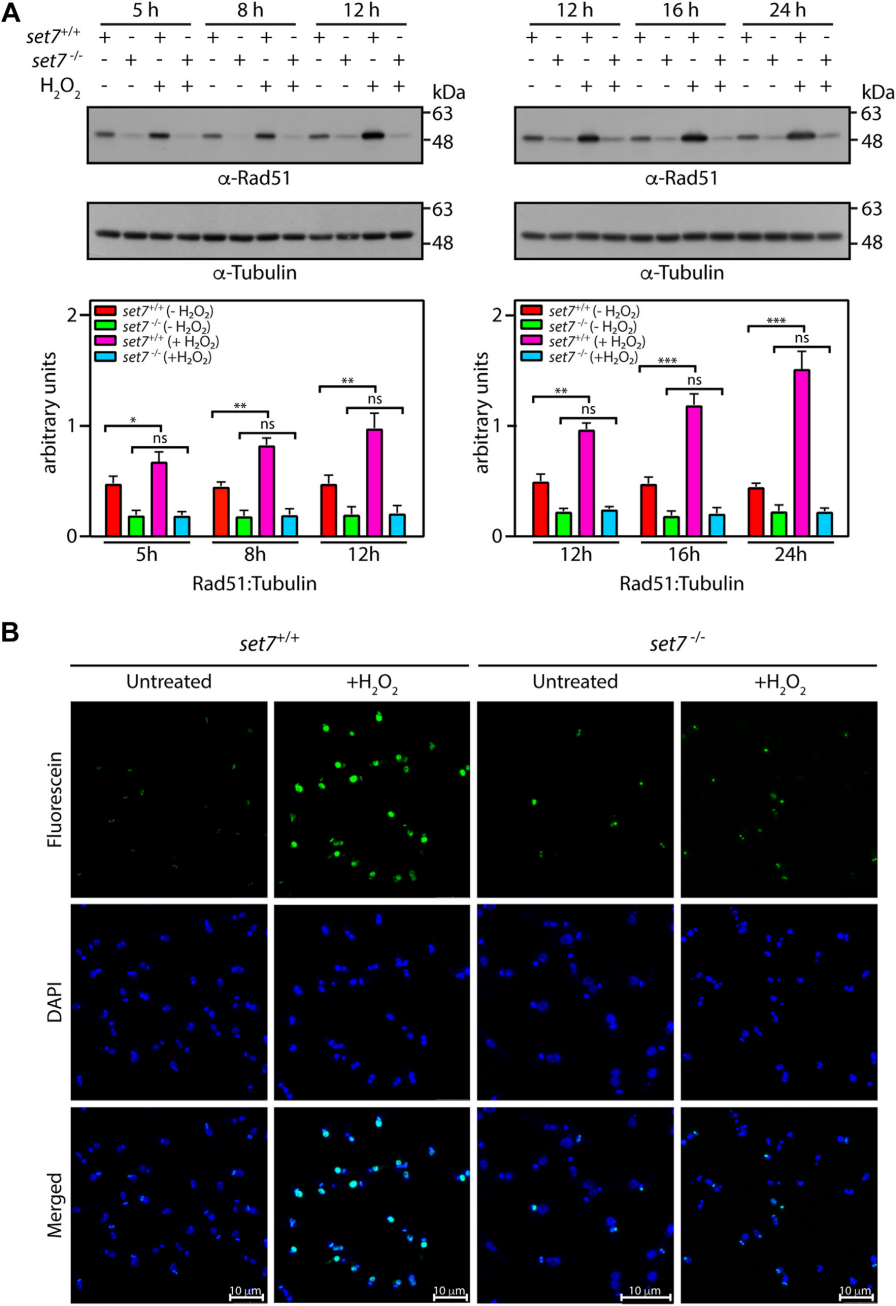
**Effect of *set7* elimination on H**_**2**_**O**_**2**_**-induced DNA damage.***A*, analysis of DNA damage response in H_2_O_2_-treated cells. Western blot analysis of whole-cell lysates, isolated from 4 × 10^7^ cells that were exposed to 100 μM H_2_O_2_ for 5 h, using anti-RAD51 antibodies (available in the lab, 1:1000 dilution). Loading control: tubulin. Time points indicate hours after start of the exposure. Quantitation was carried out using ImageJ analysis and plotted values represent average of three experiments. Error bars represent SD, and statistical significance was determined using student’s *t* test (two-tailed, unpaired). ∗∗∗*p* value <0.0005, ∗∗*p* value <0.005, ∗*p* value <0.05, and ns: statistically not significant. *B*, analysis of DNA damage in H_2_O_2_-treated cells. Microscopic analysis of TUNEL assay reactions that were performed on cells that were exposed to 200 μM H_2_O_2_ for 5 h. DAPI stains nucleus and kinetoplast in each cell. Fluorescein labels free 3′OH ends of DNA, as generated by breaks. The magnification bar represents 10 μm. Images were captured by Z-stack analysis using confocal microscopy. *set7*^*+/+*^ cells: Ld1S::neo-hyg cells. *set7*^*−/−*^ cells: *set7*-nulls. DAPI, 4′,6-diamidino-2-phenylindole; H_2_O_2_, hydrogen peroxide; Ld1S, *Leishmania donovani* 1S.

Cells in an oxidative environment experience a variety of ill effects. To determine if the discernible differential growth patterns of LdSET7-depleted cells in response to H_2_O_2_ were due to faster recovery of these cells following exposure to the oxidative agent or due to complete lack of response to H_2_O_2_ at concentrations up to 200 μM, we examined one of the consequences typically suffered by cells under these conditions: double strand DNA breaks (DSBs). H_2_O_2_-induced cellular oxidative stress leads to ssDNA and dsDNA breaks due to the production of hydroxyl free radicals (^•^OH) within the cell, which attack the bases and sugar groups in the double helix. In trypanosomatids, time-course kinetics of recruitment of various repair proteins to DSBs induced by ionizing radiation has revealed that DSBs are primarily repaired by the homologous recombination pathway involving Exo1, RPA, and RAD51 (19). RAD51-ssDNA filaments play a major role in homology recognition and strand invasion, thus promoting eventual strand exchange in the repair process, and DSBs trigger the activation of RAD51 expression and formation of distinct RAD51 foci at the DSBs 20, 21, 22. In examining the effect of H_2_O_2_ on the induction of DNA damage, we initially adopted the route of using RAD51 as a marker for DSB repair. For this, cultures of *set7*^−/−^ and *set7*^+/+^ cells were initiated from stationary phase cultures and H_2_O_2_ (100 μM) added when cells reached a density of ∼7 to 9 × 10^6^ cells/ml (*set7*^−/−^ cultures being initiated 3 days earlier to enable simultaneous H_2_O_2_ treatment of both lines), incubation carried out for 5 h, the medium replaced with fresh H_2_O_2_-free medium, and cells sampled at various time intervals thereafter for isolation of whole-cell lysates. The lysates were probed for RAD51 activation using anti-RAD51 antibodies already available in the lab. As seen in Figure 5*A*, unlike in *set7*^+/+^ parasites where RAD51 was activated over time in response to the oxidative stress induced by H_2_O_2_, in keeping with previous results from experiments with *Trypanosoma* *cruzi* (20), RAD51 levels did not increase at any of the sampled times in *set7*^−/−^ cells. Interestingly, we observed the expression of RAD51 in untreated cells also to be significantly lower in *set7*^−/−^ than *set7*^+/+^ cells. The reason for this is not understood at present and needs further exploration.

To rule out the possibility of absence of RAD51 activation in *set7*-nulls reflecting repair of DNA strand breaks occurring through a RAD51-independent pathway in these cells, we directly assessed DNA damage using the TUNEL assay, which detects DNA strand breaks by the TdT-mediated uptake of fluorescein-tagged dUMP at the free 3′OH groups generated by the breaks. Accordingly, *L. donovani* promastigotes (*set7*^+/+^ and *set7*^−/−^) were treated with H_2_O_2_ following the same regimen, for 5 h, followed by analyses for DNA breaks using the TUNEL reaction. As seen in Figure 5*B*, untreated parasites of both types (*set7*^+/+^ and *set7*^−/−^) showed labeling of kinetoplast DNA in some cells, signifying dUMP incorporation in replicating kinetoplasts (23). While hardly any nuclei were labeled in these cells, it was observed that almost twice as many *set7*^−/−^ cells exhibited nuclear labeling relative to *set7*^+/+^ cells (Table S1), suggesting that a higher fraction of these cells might carry DNA breaks, and perhaps reflecting the lower basal levels of RAD51 in *set7*^*−/−*^ cells. This aspect needs further study. Contrasting to untreated cells, *set7*^+/+^ cells displayed compelling evidence of breaks in nuclear DNA after treatment with H_2_O_2_, with more than 95% of the nuclei being strongly labelled with dUMP upon exposure to 200 μM H_2_O_2_. However, almost no evidence of damage was detectable in nuclear DNA in response to similar H_2_O_2_ treatment in *set7*^−/−^ cells (Figs. 5*B* and S7; Table S1). Taken together, the data in Figure 5 underscore the fact that *set7*^−/−^ cells do not suffer any discernible damage to nuclear DNA in response to H_2_O_2_-induced oxidative environment.

### Ectopic expression of LdSET7 in set7-nulls rescues the aberrant phenotypes associated with LdSET7 depletion

**Figure 6:**
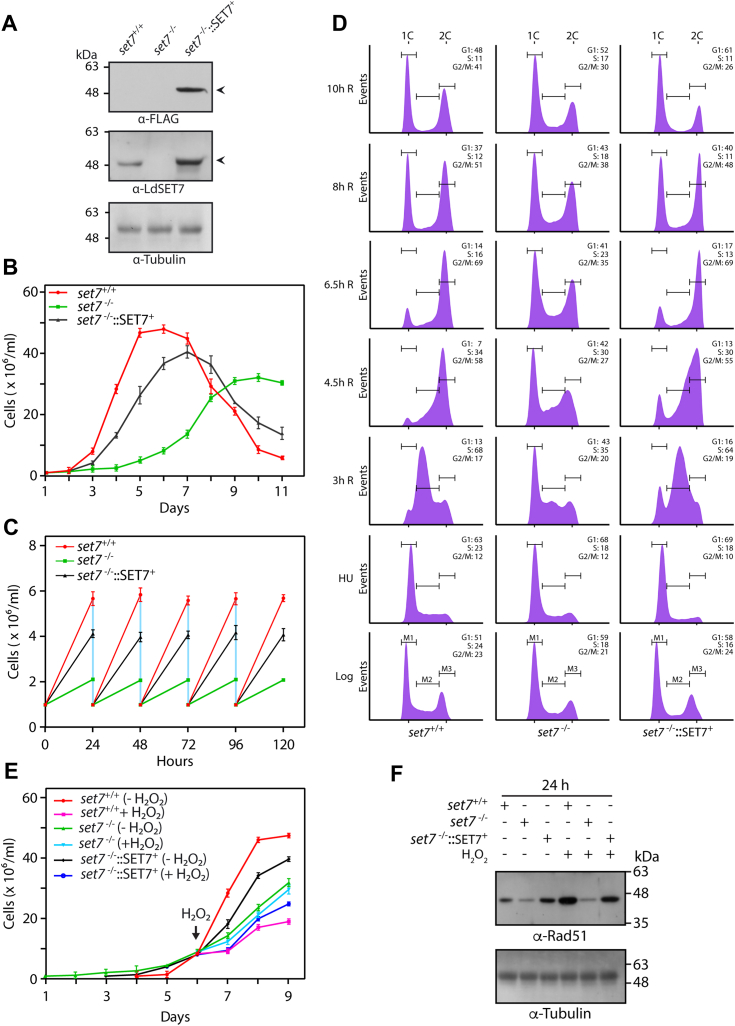
**Effect of ectopic expression of LdSET7 in *set7*-nulls, on the phenotypes associated with *set7* deletion.***A*, Western blot analysis of whole-cell extracts isolated from 8 × 10^7^ logarithmically growing promastigotes using anti-SET7 and anti-FLAG antibodies (1:1000 dilution). Loading control: tubulin. *B*, analysis of growth of parasites. Cultures were initiated at 1 × 10^6^ cells/ml, from stationary phase cultures. Values plotted represent average of three experiments, with each experiment being performed with two technical replicates. Error bars represent SD. *C*, generation time were determined by initiating cultures from logarithmically growing cultures at 1 × 10^6^ cells/ml and diluting the cultures to 1 × 10^6^ cells/ml every 24 h after counting them. Values plotted are average of three experiments, with error bars depicting SD. *D*, flow cytometry analysis of HU-synchronized promastigotes. Sampling time points are indicated on the *left* of each row of histograms. Thirty thousand events were analyzed at every time point. M1, M2, and M3 represent gating for cells in G1, S, and G2/M, respectively. Percent cells in each cell cycle phase are indicated in *upper right-hand corner* of the histograms. The experiment was carried out twice, with comparable results, and one dataset is shown here. *E*, effect of prolonged H_2_O_2_ exposure on promastigote growth. Cultures initiated from stationary phase cultures were treated with 100 μM H_2_O_2_, for 3 days, with cells being counted every 24 h. *F*, analysis of DNA damage response. Western blot analysis of whole-cell lysates was isolated from 4 × 10^7^ cells that were exposed to 100 μM H_2_O_2_ for 5 h, using anti-RAD51 antibodies (1:1000 dilution). Loading control: tubulin. Lysates were isolated 24 h after H_2_O_2_ exposure. *set7*^*+/+*^ cells: Ld1S::neo-hyg cells carrying empty vector. *set7*^*−/−*^ cells: *set7*-nulls. *set7*^*−/−*^::SET7^+^ cells: Transfectant *set7*-nulls expressing SET7-FLAG ectopically. H_2_O_2_, hydrogen peroxide; HU, hydroxyurea; Ld1S, *Leishmania donovani* 1S.

To verify that the observed phenotypes were due to LdSET7 depletion, LdSET7-FLAG was ectopically expressed in *set7*^−/−^ promastigotes as described in Experimental procedures (Fig. 6*A*) and growth of *set7*^−/−^::SET7^+^ promastigotes monitored as earlier. Ectopic expression of LdSET7 largely rescued the growth defects of *set7-*nulls (Fig. 6*B*). At ∼12 h, the generation time of *set7*^−/−^::SET7^+^ promastigotes was found to be near to that of *set7*^+/+^ cells (Fig. 6*C*). Flow cytometry analysis of HU-synchronized promastigotes revealed an alleviation of the defects observed in *set7*^−/−^ cells, upon ectopic expression of LdSET7 in them (Fig. 6*D*). The effect of H_2_O_2_-induced oxidative stress on *set7*^−/−^::SET7^+^ cells was examined by incubating cells with H_2_O_2_ (100 μM) and monitoring growth. The data in Figure 6*E* demonstrate that LdSET7-FLAG expression in *set7*-nulls allowed the parasite to largely overcome the mutant phenotype, with *set7*^−/−^::SET7^+^ promastigotes being vulnerable to H_2_O_2_ exposure almost as much as *set7*^+/+^ cells. This was also reflected in the RAD51 activation profile of *set7*^−/−^::SET7^+^ cells in response to H_2_O_2_ exposure (Fig. 6*F*). The observation of only a partial rescue of *set7*^−/−^ phenotypes could perhaps be due to differential expression of LdSET7-FLAG in the many cells of the population.

### *set7*-null parasites do not exhibit activation of reactive oxygen species in response to exposure to H_2_O_2_

**Figure 7:**
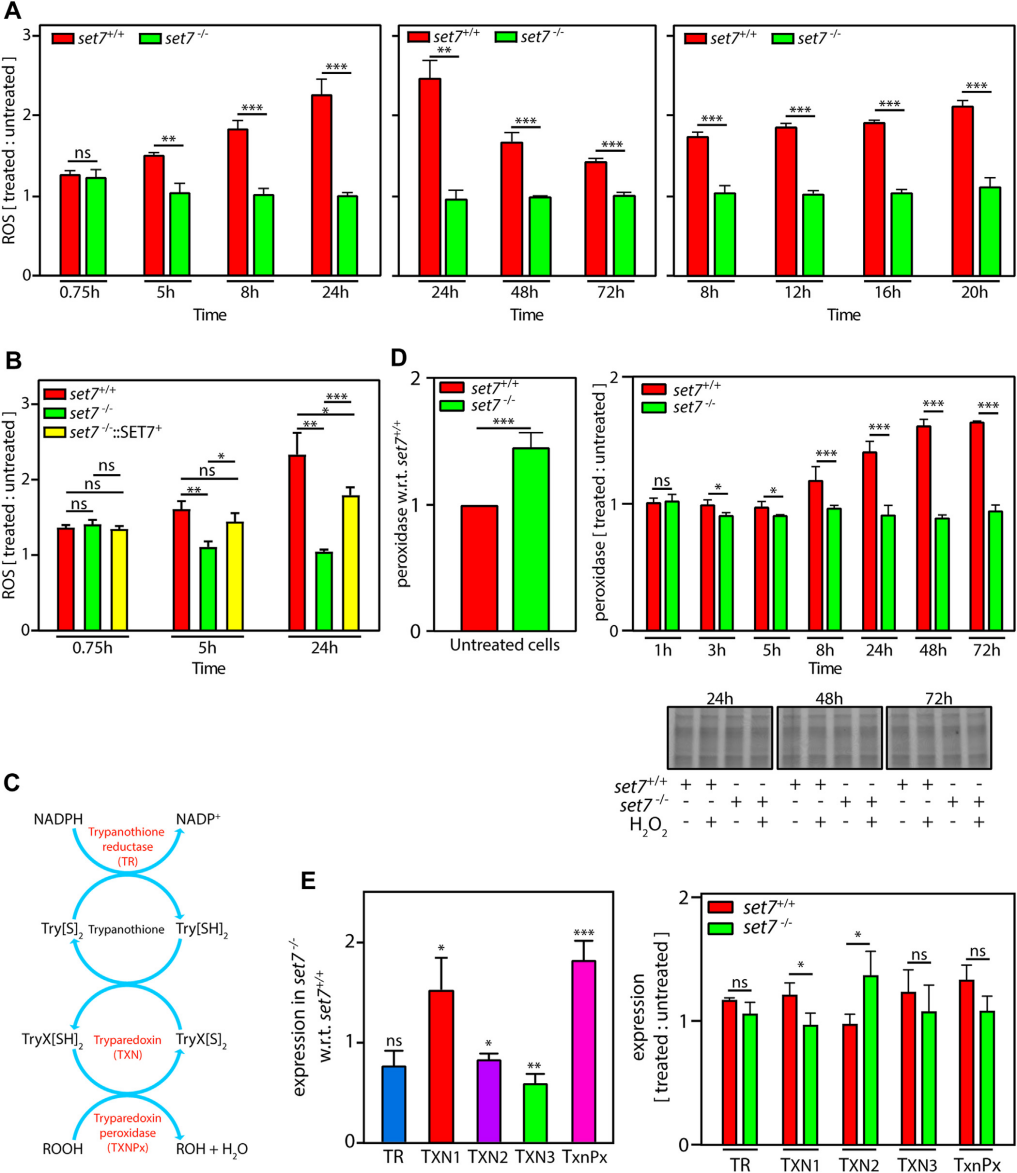
**Effect of *set7* deletion on ROS production.***A*, analysis of ROS production in *set7*^*+/+*^ and *set7*^*−/−*^ cells treated with 100 μM H_2_O_2_, relative to the respective cells which had not been exposed to H_2_O_2._ Sampling time is marked with reference to time of start of H_2_O_2_ treatment. The three graphs represent three separate experiments that were each performed thrice (as detailed in Experimental procedures), and values plotted are average of three experiments, with error bars depicting SD. Statistical significance was determined using two-tailed unpaired student’s *t* test. ∗∗∗*p* value <0.0005, ∗∗*p* value <0.005, ns: statistically not significant. *B*, analysis of ROS production in *set7*^*+/+*^, *set7*^*−/−*^*,* and *set7*^*−/−*^::SET7^+^ cells treated with 100 μM H_2_O_2_, relative to respective cells which had not been exposed to H_2_O_2._ Values plotted are average of three experiments, with error bars depicting SD. Statistical significance was determined using two-tailed unpaired student’s *t* test. ∗∗∗*p* value <0.0005, ∗∗*p* value <0.005, ∗*p* value <0.05, ns: statistically not significant *C*, schematic representation of trypanothione peroxidase scavenging system in trypanosomatids. *D*, analysis of peroxidase activity (as detailed in Experimental procedures). *Left panel*: activity in untreated *set7*^*−/−*^ cells relative to untreated *set7*^*+/+*^ cells. *Right upper panel*: activity in *set7*^*+/+*^ and *set7*^*−/−*^ cells treated with 100 μM H_2_O_2_, relative to respective cells which had not been exposed to H_2_O_2._ Sampling times are with reference to start of H_2_O_2_ treatment. *Right lower panels*: Coomassie-stained gels of cell inputs used in the reactions (input loading controls). The experiment was performed thrice (as detailed in Experimental procedures), and values plotted are average of three experiments, with error bars depicting SD. Statistical significance was determined using two-tailed unpaired student’s *t* test. ∗∗∗*p* value <0.0005, ∗*p* value <0.05, ns: statistically not significant. *E*, real-time PCR analysis of transcripts of the trypanothione peroxidase system. Fold difference in expression was determined using 2^−▵▵Ct^ method. Tubulin served as internal control for each RNA sample. *Left panel*: expression in untreated *set7*^*−/−*^ cells with reference to untreated *set7*^*+/+*^ cells. For each cell type, the Ct values of the genes and of tubulin were determined, from these the ▵Ct values (Ct of gene − Ct of tubulin) obtained, and thereafter the ▵▵Ct values (▵Ct of *set7*^−/−^ − ▵Ct of *set7*^+/+^) were calculated. The *bar graphs* represent the 2^−▵▵Ct^ values (fold change). *Right panel*: expression in H_2_O_2_ -treated *set7*^*+/+*^ and *set7*^*−/−*^ cells with reference to the respective untreated cells. For each cell type and each condition (untreated and H_2_O_2_-treated), the Ct values of the genes and of tubulin were determined, and from these the ▵Ct values (Ct of gene − Ct of tubulin) obtained. Thereafter, for each cell type, the ▵▵Ct values (▵Ct of treated cells − ▵Ct of untreated cells) were obtained, and the 2^−▵▵Ct^ values calculated. The *bar graphs* represent the 2^−▵▵Ct^ values (fold change in expression). Data plotted are average of three experiments, each experiment being performed with technical duplicates. *Bar graphs* represent 2^−▵▵Ct^ values. Error bars signify SD. Statistical significance was assessed using two-tailed unpaired student’s *t* test. ∗∗∗*p* value <0.0005, ∗∗*p* value <0.005, ∗*p* value <0.05, and ns: statistically not significant. H_2_O_2_, hydrogen peroxide; ROS, reactive oxygen species; TR, trypanothione reductase; TXN1, tryparedoxin 1; TXN2, tryparedoxin 2; TXN3, tryparedoxin 3; TxnPx: tryparedoxin peroxidase.

The data in Figure 2, Figure 3, Figure 4, Figure 5, Figure 6 strongly indicated that LdSET7 depletion modulates the cell’s response to an oxidizing environment. Particularly intriguing in *set7*-nulls was the apparent absence of detectable DNA damage, expected to be induced by the production of reactive oxygen species (ROS) upon exposure to H_2_O_2_ (Fig. 5). The production of ROS in *set7*^*−/−*^ cells was compared with that in *set7*^*+/+*^ cells using the dichlorodihydrofluorescein diacetate (DCFDA) assay (24). This assay is based on the principle that DCFDA taken up by cells is deacetylated by cellular esterases to dichlorodihydrofluorescein. Intracellular ROS oxidize the dichlorodihydrofluorescein to dichlorofluorescein, whose fluorescence is measured spectrofluorimetrically (488 ex/529 em). The assay was carried out by incubating *set7*^+/+^ and *set7*^−/−^ promastigotes in medium carrying H_2_O_2_ (100 μM), before incubation with DCFDA and analysis by measurement of fluorescence emission at 529 nm (as detailed in Experimental procedures). It was observed that while ROS levels increased in *set7*^+/+^ cells over 45 min to 24 h after exposure to H_2_O_2_ before gradually dropping, no appreciable change in ROS (in comparison with untreated cells) was detectable in *set7*^−/−^ cells over the same period of time except a slight elevation detected just after exposure to H_2_O_2_ (Fig. 7*A*). Ectopic expression of LdSET7 in *set7*^−/−^ parasites partially rescued the mutant phenotype (Fig. 7*B*). The apparent absence of induction of ROS production in response to H_2_O_2_ treatment in *set7*^−/−^ cells may be the likely reason for these cells not suffering DNA damage under these conditions and could also explain why the parasites are apparently resistant to fairly high concentrations of H_2_O_2._ This observation also points toward the possibility of a significantly higher efficiency of ROS scavenging in *set7*^−/−^ parasites.

Whereas the mammalian defense against oxidative stress is largely glutathione-dependent, with intracellular superoxide radicals being converted to molecular oxygen and H_2_O_2_ by superoxide dismutase, and H_2_O_2_ in turn being converted to water and oxygen by enzymes like glutathione peroxidase and catalase, trypanosomatids employ a somewhat different modus operandi. Lacking catalase and selenium-dependent peroxidases, instead, they possess a unique trypanothione-dependent system with a set of enzymes that act concertedly for the detoxification of peroxides. The three main components of this system are trypanothione reductase (TR), tryparedoxin (TXN), and tryparedoxin peroxidase (TxnPx) (Fig. 7*C*). Of the three TxnPxs harbored by the parasite, only one is cytosolic and the cytosolic TxnPx (cTxnPx) is the primary mediator of hydroperoxide scavenging in the macrophage environment. While TR reduces the thiol trypanothione (TS_2_) to T(SH)_2_ using NADPH, TXN (a thiol disulphide oxidoreductase) serves as the conduit through which the reducing equivalents flow from T(SH)_2_ to cTxnPx (a 2-Cys peroxiredoxin), and the reduced cTxnPx reacts with and catalyzes the breakdown/reduction of hydroperoxides. The absence of detectable ROS induction in response to H_2_O_2_ treatment led us to consider if this trypanothione-dependent system was overactive in *set7*^*−/−*^ cells. Thus, we examined peroxidase activity in *set7*^*−/−*^ parasites, as described in Experimental procedures. Results of activity assays revealed that untreated *set7*^*−/−*^ parasites possessed significantly higher peroxidase activity relative to untreated *set7*^*+/+*^ parasites (Fig. 7*D*, left panel). Interestingly, while peroxidase activity was enhanced in *set7*^*+/+*^ parasites over 1 h to 72 h after exposure to H_2_O_2_, *set7*^*−/−*^ parasites did not demonstrate any visible change in peroxidase activity in response to H_2_O_2_ over the same period, suggesting that elimination of LdSET7 was causing loss of regulation of peroxidase activity, and the higher levels of basal peroxidase activity in *set7*^*−/−*^ cells were sufficient to rapidly scavenge ROS (Fig. 7*D*).

In contemplating the reasons for enhanced basal peroxidase activity in *set7*-nulls, the possibility of higher expression of TXN peroxidase was considered. We were unable to check protein expression levels directly in the absence of available antibodies. Thus, we analyzed transcript levels of the enzyme, as well as of the other components of the trypanothione-dependent peroxide detoxification system, in *set7*^*−/−*^ cells in comparison with *set7*^+/+^ cells. This was done by real-time PCR analysis of RNA isolated from untreated and H_2_O_2_-treated (8 h after start of the 5 h-treatment with 100 μM H_2_O_2_) cells. On comparing expression in *set7*^*−/−*^ and *set7*^+/+^ untreated cells, it was observed that cTxnPx was expressed almost 2-fold higher in these cells (Fig. 7*E*, left panel). In response to H_2_O_2_ treatment, none of the transcripts were significantly elevated in either type of parasite (Fig. 7*E*, right panel). The enhanced peroxidase activity in *set7*^*+/+*^ cells in response to H_2_O_2_ exposure (Fig. 7*D*) reflects previous results from Iyer *et al.*, who reported activated expression of the TXN peroxidase protein in *L. donovani* in response to H_2_O_2_ treatment (25); our data indicates that this is not due to elevated transcript levels.

The data in Figure 7, *D* and *E* suggest that LdSET7 regulates peroxidase activity by controlling cTxnPx expression: deletion of *set7* is coupled to significantly higher levels of *txnPx* transcripts in the cells regardless of exposure to an oxidative environment, in turn resulting in higher peroxidase activity which probably leads to rapid scavenging of the ROS produced in the cell in response to H_2_O_2_ treatment, such that ROS activation escapes detection and the effects of ROS activation (such as DNA damage) are not experienced.

### Ectopic expression of LdSET7-Y421A in *set7*-nulls does not rescue the deviant phenotypes associated with SET7 depletion

**Figure 8:**
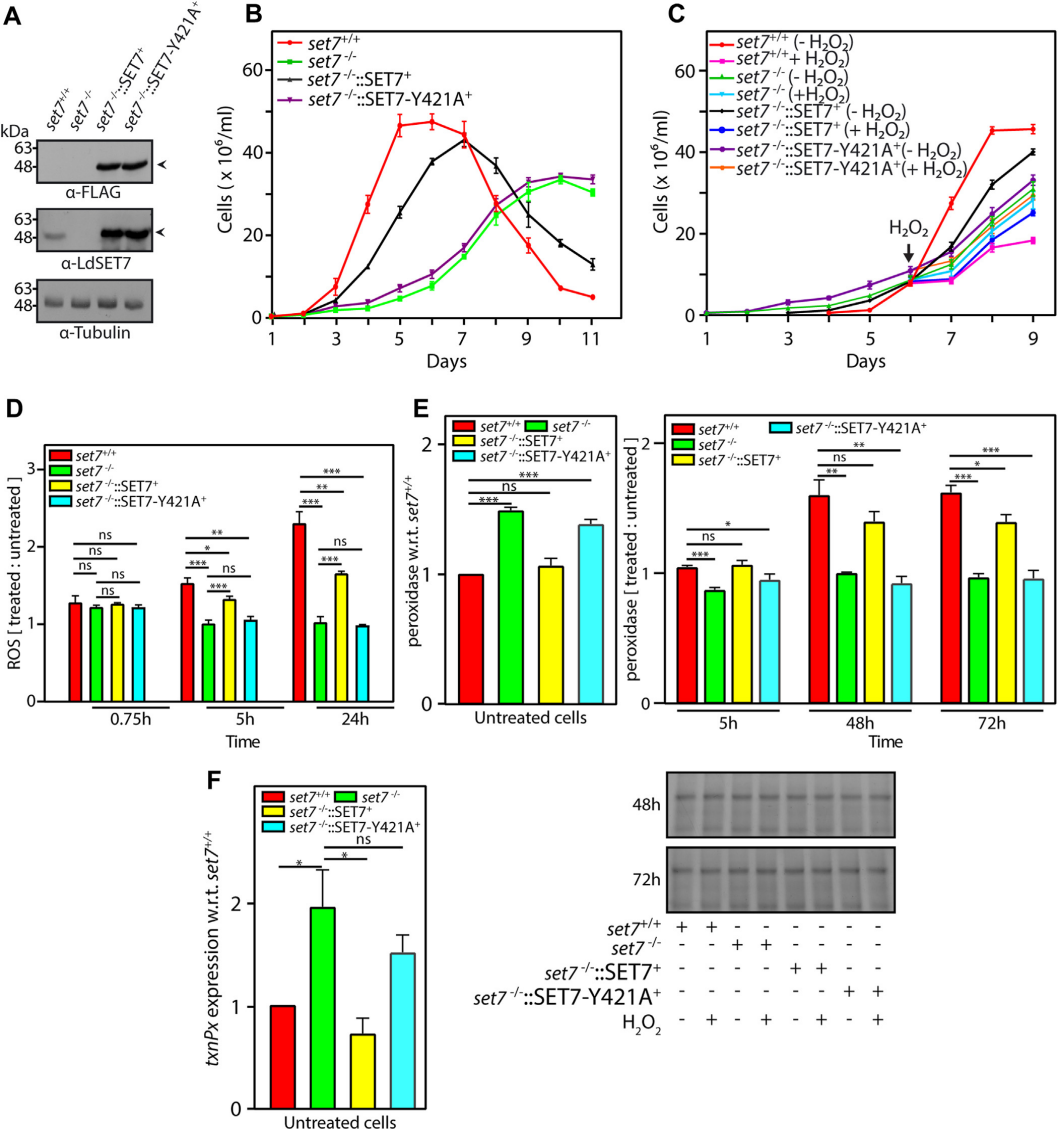
**Effect of ectopic expression of LdSET7-Y421A in *set7*-nulls, on the phenotypes associated with *set7* deletion.***A*, Western blot analysis of whole-cell extracts isolated from 8 × 10^7^ logarithmically growing promastigotes using anti-SET7 and anti-FLAG antibodies (1:1000 dilution). Loading control: tubulin. *B*, analysis of growth of parasites. Cultures were initiated at 1 × 10^6^ cells/ml from stationary phase cultures. Values plotted represent average of three experiments, with each experiment being performed with two technical replicates. Error bars represent SD. *C*, effect of prolonged H_2_O_2_ exposure on promastigote growth. Cultures initiated from stationary phase cultures were treated with 100 μM H_2_O_2_, for 3 days, with cells being counted every 24 h. *D*, analysis of ROS production in *set7*^*+/+*^, *set7*^*−/−*^, *set7*^*−/−*^::SET7^+^, and *set7*^*−/−*^::SET7-Y421A^+^ cells treated with 100 μM H_2_O_2_, relative to respective cells which had not been exposed to H_2_O_2._ Values plotted are average of three experiments, with error bars depicting SD. Statistical significance was determined using two-tailed unpaired student’s *t* test. ∗∗∗*p* value <0.0005, ∗∗*p* value <0.005, ∗*p* value <0.05, and ns: statistically not significant. *E*, analysis of peroxidase activity. *Left panel*: activity in untreated *set7*^*−/−*^*, set7*^*−/−*^::SET7^+^, and *set7*^*−/−*^::SET7-Y421A^+^ cells, relative to untreated *set7*^*+/+*^ cells. *Right upper panel*: activity in *set7*^*+/+*^, *set7*^*−/−*^, *set7*^*−/−*^::SET7^+^, and *set7*^*−/−*^::SET7-Y421A^+^ cells treated with 100 μM H_2_O_2_, relative to the respective cells which had not been exposed to H_2_O_2._ Sampling times are with reference to start of H_2_O_2_ treatment. *Right lower panels*: Coomassie-stained gels of cell inputs used in the reactions (input loading controls). The experiment was performed thrice, and values plotted are average of three experiments, with error bars depicting SD. Statistical significance was determined using two-tailed unpaired student’s *t* test. ∗∗∗*p* value <0.0005, ∗∗*p* value <0.005, ∗*p* value <0.05, ns: statistically not significant. *F*, real-time PCR analysis of *txnPx* transcripts: expression in untreated *set7*^*−/−*^/WT complement/Y421A complement cells with reference to untreated *set7*^*+/+*^ cells. Fold difference in expression was determined using 2^−▵▵Ct^ method. Tubulin served as internal control for each RNA sample. For each cell type, the Ct values of the *txnPx* gene and of tubulin were determined, from these the ▵Ct values (Ct of *txnPx* − Ct of tubulin) obtained, and thereafter the ▵▵Ct values (▵Ct of *set7*^−/−^/WT complement/Y421A complement − ▵Ct of *set7*^+/+^) were calculated. The *bar graphs* represent the 2^−▵▵Ct^ values (fold change). *set7*^*+/+*^ cells: Ld1S::neo-hyg cells carrying empty vector. *set7*^*−/−*^ cells: *set7*-nulls. *set7*^*−/−*^::SET7^+^ cells: transfectant *set7*-nulls expressing SET7-FLAG ectopically. *set7*^*−/−*^::SET7-Y421A^+^ cells: transfectant *set7*-nulls expressing SET7-Y421A-FLAG ectopically. H_2_O_2_, hydrogen peroxide; ROS, reactive oxygen species.

The structure of the SET domain is largely loops and turns, with several small β sheets carrying a few short strands (26). The C-terminal end of the SET domain carries a short conserved stretch of residues harboring an invariant tyrosine residue. This stretch is preceded by a pseudoknot structure. The pseudoknot and invariant tyrosine residue are essential for AdoMet binding and catalysis, with the conserved tyrosine playing a critical role in catalysis. Mutation of this tyrosine residue has been linked to loss of the protein’s methylation activity (27). The data in Figure 1, Figure 2, Figure 3, Figure 4, Figure 5, Figure 6, Figure 7 suggest that LdSET7 plays a role in moderating the cell’s response to oxidative stress. To determine if LdSET7 methylation activity is critical to this function, we resorted to mutating this conserved tyrosine residue of LdSET7 (Fig. S1; mutagenesis detailed in Experimental procedures). The SET7-Y421A-FLAG protein was expressed ectopically in *set7*^−/−^ promastigotes (Fig. 8*A*) and when the growth pattern of *set7*^−/−^:: SET7-Y421A^+^ promastigotes was monitored it was found to resemble that of *set7*^−/−^ cells (Fig. 8*B*). *set7*^−/−^::SET7-Y421A^+^ promastigotes displayed a response similar to *set7*-nulls when exposed to H_2_O_2_ as well (Fig. 8*C*). Analyses of ROS production (Fig. 8*D*), peroxidase activity (Fig. 8*E*), and *txnPx* transcript levels (Fig. 8*F*) revealed that ectopic expression of the SET7-Y421A protein in *set7*-nulls could not rescue the phenotypes associated with *set7* deletion, underscoring the significance of this residue in LdSET7 function, and implicating a role for LdSET7 methylation activity in moderating the cell’s response to oxidative stress.

## Discussion

As the cause of Visceral Leishmaniasis, the mechanisms by which *L. donovani*’s physiological processes are regulated remain an area of intensive research. Gene regulation is somewhat unusual in *L. donovani* and other trypanosomatids, with the genes being organized in long unidirectional clusters of functionally unrelated genes that are by-and-large coordinately and constitutively transcribed polycistronically, from divergent strand switch regions, with the *trans-*splicing of short leader sequences at the 5′ ends of the individual gene units and polyadenylation at the 3′ ends of these units, leading to the production of individual mRNAs ready for translation. No consensus sequences or defined promoter elements have been recognized at these TSRs, nor have the wide repertoire of canonical transcriptional activators found in more conventional eukaryotes, been identified as yet in these organisms. Epigenetic mechanisms appear to control gene expression in trypanosomatids to a fair extent, and the roles of specific histone acetylations in modulating transcription and other DNA-related processes in *Leishmania* as well as *Trypanosoma* species have been documented (28, 29, 30, 31, 32, 33, 34). The functions of histone methylations and the proteins mediating them, however, remain largely unexplored in these organisms, with studies thus far revealing the enrichment of H3K4 methylation marks at TSRs (34, 35), and the role of the *Trypanosoma* Dot1 proteins in modulation of DNA replication (*via* H3K76 methylation) being uncovered (36). Histone methylations at lysine residues are mediated by SET-domain proteins (Dot1 proteins being the only other histone lysine methyltransferases), and while 29 SET-domain proteins have been identified in *T. brucei*, all of which are conserved in *L. donovani*, their target substrates have not been identified yet, and the cellular role of only one of them (TbSET27) has been elucidated thus far (11, 14). SET proteins that have been extensively studied across eukaryotes, particularly yeast and mammalian cells, have been found to target non-histone substrates as well (1, 37), highlighting the fact that they regulate cellular processes through a wide range of downstream substrates. In several instances one or more functions of a SET protein have been unearthed without the target substrate(s) being identified. These proteins often display stringent catalytic activity as they may mediate monomethylation, dimethylation, or trimethylation of the target lysine residue, translating into tighter regulation of the pertinent cellular process as the extent of methylation of a particular lysine residue may govern protein–protein interactions. SET protein–mediated methylations also impact protein activity and protein stability.

In investigating the LdSET7 protein, we found the primarily cytosolic LdSET7 was expressed in promastigotes throughout the cell cycle (Figs. S2 and S3). LdSET7 was not essential to the parasite, but *set7*^*−/−*^ promastigotes exhibited slower growth and a heightened sensitivity to HU-induced G1/S arrest in comparison to *set7*^*+/+*^ cells (Fig. 3). Contrastingly, *set7*^*−/−*^ amastigotes survived more proficiently than *set7*^*+/+*^ amastigotes within host macrophages (Fig. 4*A*), indicating that LdSET7 plays a role in moderating the parasite’s response to the hostile intracellular oxidative environment of host cells. Interestingly, this effect was tightly controlled by LdSET7 expression levels: partial depletion of LdSET7 to ∼50% its usual expression, while not having any effect on growth or cell cycle progression of promastigotes (Figs. 1*C* and S4), led to much higher proficiency of survival of amastigotes in host macrophages (Fig. 1*D*), even higher than that of *set7*^*−/−*^ amastigotes (Fig. 4*A*). The impact of LdSET7 depletion on amastigote growth and survival was mirrored in the response of promastigotes to an oxidative growth environment *in vitro*, wherein partial depletion of LdSET7 allowed the parasites to tolerate H_2_O_2_-induced oxidative stress better than usual (Fig. 2), while complete elimination of LdSET7 made the parasites almost completely resistant to stress induced by H_2_O_2_ at concentrations up to 200 μM (Fig. 4, *B* and *C*). The resistance of *set7*^*−/−*^ promastigotes to H_2_O_2_-induced stress was apparent not only in the growth kinetics of the parasites but also in the almost complete absence of DNA damage in response to H_2_O_2_ exposure (Figs. 5 and S7; Table S1). ROS-induced stress in mammalian cells is known to alter the subcellular localization of the p65/RelA subunit of the NF-kB transcription protein complex, relocating the cytoplasmic protein to the nucleus where it exercises its ability to activate transcription of several genes such as Mn-SOD and catalase (37, 38). No obvious alteration in the subcellular localization of LdSET7 was detectable in response to H_2_O_2_ exposure (Fig. S6), and LdSET7 expression was not upregulated upon H_2_O_2_ exposure either (Fig. 4*D*). The finding that LdSET7 elimination makes the parasite resistant to H_2_O_2_-induced oxidative stress and allows amastigotes to survive more proficiently suggests that the parasite (WT) harmonizes its response with the host environment, to withstand the onslaught of the host defense system and proliferate in an orderly fashion, thus establishing itself firmly in the host. The higher survival and proliferation rates associated with *set7* deletion would lead to a depletion of host cells with time, thence being detrimental to the persistence of infection.

Amastigotes express a gamut of proteins to tolerate and overcome the stress induced by the oxidative environment of the macrophages. While the macrophages assault the parasites early in infection by releasing a burst of ROS, the parasite employs a barrage of proteins to fight the ROS wave. These include antioxidant proteins and enzymes to scavenge the ROS, as well as proteins to repair DNA lesions induced by the oxidative environment. Hydroperoxides in *Leishmania* are scavenged by a trypanothione-dependent system. Three TXN genes have been annotated in the *L. donovani* genome and of the three TxnPxs that have been identified in *Leishmania*, one is cytosolic (cTxnPx: LdBPK_151140.1) and essential for cell survival, the second is mitochondrial (mTxnPx: LdBPK_230050.1), and the other is glycosomal (gTxnPx:LdCL_150016500) (25, 39). The cytosolic TxnPx is the primary mediator of hydroperoxide scavenging within host macrophages and has been reported to be secreted in *Leishmania infantum* and *Leishmania major* (40). The data in Figure 7 suggests that LdSET7 controls expression and activity of TxnPx through transcript levels (Fig. 7, *D* and *E*), with *set7* deletion leading to rapid scavenging of any ROS generated (Fig. 7*A*). Moreover, the observations of experiments carried out with the LdSET7-Y421A protein (Fig. 8) strongly suggest that SET7-mediated methylation activity is critical to the protein’s impact on host–parasite interactions.

While the mechanism by which LdSET7 controls *txnPx* transcript levels remains unknown to us, two general possibilities are contemplatable. In the first scenario, LdSET7 may regulate *txnPx* transcripts either through an epigenetic mark on a histone residue, which would have an impact on global gene expression or through the methylation of a transcription factor which downregulates *txnPx* transcription. Considering that LdSET7 is predominantly cytosolic, it is unlikely to mediate an activating/repressive histone methylation mark, which would usually be added after histone deposition. While *txnPx* transcripts are upregulated in *set7*-nulls (Fig. 7*E*), other genes lying in the same polycistronic cluster remain unaffected by *set7* deletion in untreated as well as H_2_O_2_-exposed cells (data not shown), indicating that LdSET7 does not modulate global gene activation/repression. Contemplating the alternate possibility of LdSET7 controlling the expression of *txnPx* transcripts through the methylation of a transcriptional activator: previous studies have reported SET protein–mediated methylation to impact activity of such proteins. Intracellular ROS accumulation in mammalian cells shuttles the p65/RelA subunit of the NF-kB transcription factor into the nucleus, ultimately triggering activation of genes whose products fight the oxidative burst. While Set7/9-mediated methylation of p65/RelA at a specific lysine residue is believed to be critical to RelA’s ability to activate transcription, methylation of RelA at other lysine residues by the same SET protein has been reported to repress its ability to activate transcription (reviewed in (37)). The abundantly cytosolic human SET protein SMYD2 methylates p53, which shuttles between the nucleus and cytoplasm, and this methylation (at K370) inhibits p53 transactivation activity (41). Thus, LdSET7-mediated methylation of a transcriptional activator may inhibit its activity, tightly controlling *txnPx* transcript levels, and deletion of *set7* may alleviate this negative regulatory effect. While no evidence in support of gene regulation through transcriptional activators has been uncovered in trypanosomatids thus far, with transcription being primarily polycistronic and constitutive, a small subset of genes have been identified to be transcriptionally activated in a cell cycle–dependent manner. These genes are scattered throughout the genome and are turned on by promoters immediately upstream of them (28). It is possible that *txnPx* is activated by a promoter of this kind; this needs to be further investigated.

In the second scenario, in light of the fact that posttranscriptional processes are crucial to regulating gene expression in these organisms, the roles of RNA-binding proteins (RNA-BPs or RBPs) and/or RNA methylation may be a vital factor. 5′ and 3′ UTRs of stable processed transcripts often carry *cis*-acting elements that serve as regulatory motifs to which RBPs bind. The RBP–RNA interactions modulate mRNA stability, decay, transport, and can also modulate translation efficiency. The *Trypanosoma cruzi* UBP1 protein has been reported to bind to a specific sequence UBP1m in the 3′UTR of the gene transcripts whose expression it regulates (mostly those encoding surface glycoproteins), thus stabilizing the mRNAs (42). Two yeast SET proteins have themselves been found to be RBPs, with evidence of RNA binding activity *in vitro* as well as *in vivo* (43, 44). These proteins carry RNA recognition motifs (RRMs) and are enriched on only a small number of transcripts, with the SET–mRNA interactions being hypothesized to contribute toward holding the SET proteins in proximity with their target histone residues. Although it is possible that LdSET7 may itself be an RBP, no RRM has been identifiable. In *Trypanosoma brucei*, while almost 350 transcripts have been found to be enriched in N^6^-methyladenosine, with RNA methylation in the poly(A) tails of VSG transcripts being found to stabilize VSG mRNAs, *txnPx* transcripts are not among those identified with N^6^-methyladenosine marks thus far (45, 46). Considering also that to date there is no evidence of SET proteins being RNA methylases and LdSET7-mediated methylation activity appears to be critical to modulating *txnPx* transcript levels (Fig. 8*F*), it seems unlikely that LdSET7 is directly controlling *txnPx* transcript levels. It is possible that LdSET7 mediates the methylation of an RNA methylase that targets *txnPx* transcripts, with SET7-mediated methylation having an inhibitory effect on activity; these possibilities need further in-depth studies.

While PTMs on proteins have been widely studied and are generally known to regulate protein function through their impact on localization, protein–protein interactions, stability, and activity, not much information is currently available on the impact of PTMs in case of RNA-BPs. In *Saccharomyces cerevisiae*, the Set3 protein methylates Nab3 of the Nrd1–Nab3–Sen1 complex, and mutation of the target lysine residue which lies in its RRM leads to reduced binding to RNA *in vitro* and transcription termination defects *in vivo* (47). An in-depth analysis of the PTM landscape on RBPs in human cells has been carried out through collation of existing curated datasets and published research (48). PTMs have been identified in almost 2400 RBPs, with the vast majority of these carrying multiple PTMs. The commonest classes of PTMs identified were phosphorylation, acetylation, ubiquitination, and methylation. Methylations on RBPs have been found in over 1100 proteins and while typically occurring at arginine residues, methylation of RBPs at lysine residues have also been identified in several human cancer cell lines as well as in clinical samples drawn from different body organs (48). Thus, it is possible that LdSET7 mediates its effect through the methylation of one or more RBPs, the negative impact of which would be reflected in tightly controlled levels of target transcripts, in this case, *txnPx.* Depletion of LdSET7 and corresponding abrogation of the RBP methylation event would be coupled to loss of this regulation, resulting in an overall increase in *txnPx* transcript expression levels. The mammalian SET7/9-mediated E2F1 methylation marks it for degradation *via* the ubiquitination–proteasomal degradation pathway (49); a similar mechanism could gradually deplete the parasite of an RNA-BP that is vital to stabilizing *txnPx* transcripts.

This study reports the first data directly investigating the functional role of a SET protein in *Leishmania* species, with the results of our experiments demonstrating that LdSET7 modulates the persistence of *Leishmania* infection by working toward maintaining the delicate balance between parasite and host cell. The distinct phenotypes obtained upon *set7* deletion suggest that although 29 SET proteins have been identified in these organisms, functional redundancy may be limited. While the global landscape of histone modifications has not been uncovered yet in *Leishmania* species, a large number of methylation events have been identified in *T. brucei* and *T. cruzi* histones, and several of these SET proteins must target histone substrates. The identification of target histone substrates of specific SET proteins will allow us to gain new insights into how these proteins function.

## Experimental procedures

### *Leishmania* cultures and manipulations

Ld1S cultures were grown in M199 medium supplemented with fetal bovine serum (Invitrogen), adenine, and hemin (Sigma Aldrich), at 26 °C as described earlier (50). Growth and survival patterns were analyzed as described earlier (30). Generation time in the logarithmic phase of growth was determined as described earlier (28). Synchronization of parasites using HU (5 mM for 8 h) and flow cytometry analyses were carried out as described previously (18). Transfections were done and clonal lines generated as described (29, 30). Whole-cell extracts were isolated using the M-PER kit (Thermo Fisher Scientific) as per manufacturer’s instructions. Procylics and metacyclics were isolated as described earlier (51). For treatment with H_2_O_2_, cultures were initiated from stationary phase cultures, at 1 × 10^6^ cells/ml, and H_2_O_2_ (25–1000 μM) added on reaching a cell density of ∼7 to 9 × 10^6^ cells/ml (day 3 for *set7*^+/+^ and *set7*^−/+^ cells, day 6 for *set7*^−/−^ cells). The *set7*^−/−^ cultures were initiated 3 days earlier to enable addition of H_2_O_2_ at the same time as the *set7*^*+/+*^ and *set7* ^−/+^ cells. At the time of addition of H_2_O_2_, the cultures were split into two, with half the cells receiving H_2_O_2_ treatment and the other half being carried forward as the untreated culture. The cultures were incubated further at 26 °C for varying time intervals and sampled for further experimentation.

### Cloning of *set7* gene for expression in *Leishmania*

For expression in *Leishmania* parasites, the pLEXSY-FLAG vector was created from the pLEXSY-CYC9-FLAG plasmid (28) by digesting it with BglII (which would drop out the CYC9 gene but not the FLAG sequence it is tagged with) and ligating the vector’s BglII ends. For expressing the *set7* gene in *L. donovani* promastigotes, the gene was amplified off Ld1S genomic DNA using primers SET7-FLAG-F (5′-TAGGATCCATGCCCATCAGCCAG-3′) and SET7-FLAG-R (5′- TAGGATCCAGGAAGAAGAGGCTTCA-3′). The amplicon was cloned into the BglII site of the pLEXSY-FLAG vector using the BamHI sites in the primers, creating plasmid pLEXSY/SET7-FLAG. The SET7*-*Y421A mutation was created using overlap PCR. For this, the N-terminal fragment of the gene was amplified using primers SET7-FLAG-F and SET7-Y421A-R (5′-AAAGGTGGCGGCGTCCATGCTCAAGT-3′), while the C-terminal fragment was amplified using primers SET7-Y421A-F (5′- ACTTGAGCATGGACGCCGCCACCTTT-3′) and SET7-FLAG-R. The full-length amplicon was obtained using a mix of the two amplicons as template, with the help of primers SET7-FLAG-F and SET7-FLAG-R. The amplicon carrying the mutation was cloned into the BglII site of the pLEXSY-FLAG vector, creating plasmid pLEXSY/SET7-Y421A-FLAG.

### Creation of knockout and rescue lines

The first *set7* genomic allele was knocked out by replacing it with a *hyg*^*r*^ cassette, while the second allele was replaced with a *neo*^*r*^ cassette. The donor plasmid for replacing the second allele was constructed using the pLEXSY_I-neo3 vector as backbone (Jena Bioscience). To replace the first allele, first the *neo*^*r*^ cassette in pLEXSY_I-neo3 was replaced with a *hyg*^*r*^ cassette using the BamHI-SpeI sites flanking the *neo*^*r*^ cassette, generating the vector pLEXSY/hyg, which served as the backbone for constructing the donor plasmid. The ∼800 bp region immediately upstream of the *set7* gene was amplified using Ld1S genomic DNA with primers SET7-5′FL-F (5′- TAGCGGCCGCATTTAAATGGTTTTTTCTGCCTTCTCTTG-3′) and SET7-5′FL-R (5′- TAGCGGCCGCGGTCTGCGACCGATATACCTCGG-3′) and the amplicon cloned into the NotI site of the two vector backbones. The ∼ 800 bp region immediately downstream of the *set7* gene was amplified using primers SET7-3′FL-F (5′- TAACTAGTATACATGAGTGAGACGCTCCGCGG-3′) and SET7-3′FL-R (5′- TAACTAGTATTTAAATTGCTTCTCAAAGCCCTTGTCA-3′) and the amplicon cloned into the SpeI site of the two clones carrying the 5′flank sequence, thus creating the donor plasmids SET7-KO/hyg and SET7-KO/neo.

For making the *set7* heterozygous knockout (*set7*^−/+^) the donor cassette was released from plasmid SET7-KO/hyg using SwaI digestion, transfected into *L. donovani* promastigotes by electroporation, and clonal lines selected for and expanded in the presence of hygromycin (16 μg/ml), as described earlier (29). For making the *set7*-null (*set7*^−/−^) the donor cassette was released from plasmid SET7-KO/neo using SwaI digestion, transfected into *set7*^−/+^ promastigotes by electroporation, and clonal lines selected for and expanded in the presence of G418 (50 μg/ml) and hygromycin (16 μg/ml). Clonal lines were maintained in liquid culture under selection pressure (G418 at 100 μg/ml and hygromycin at 32 μg/ml) except for flow cytometry experiments where the drugs were withdrawn a week before setting up the experiment.

For making the rescue line, the *set7* gene was cloned into the BamHI-EcoRV sites of pXG-FLAG (bleo) vector (30), using primers SET7-pXG-F (5′-GAGGATCCGCCACCATGCCCATCAGCCAG-3′) and SET7-pXG-R (5′-AGGATATCTCCAGGAAGAAGAGGCTT-3′) to amplify the gene for cloning. The plasmid pXG-SET7-FLAG (bleo) was transfected into *set7*^−/−^ promastigotes and clonals selected for using G418, hygromycin, and phleomycin (50 μg/ml, 16 μg/ml and 2.5 μg/ml, respectively).

### Isolation of RNA and real-time PCRs

Total RNA was isolated from 5 × 10^7^ promastigotes with the help of the PureLink RNA mini kit (Invitrogen). The RNA was treated with DNaseI (1 U DNase I per 2 μg RNA at 37 °C for 30 min) prior to cDNA synthesis, to eliminate any genomic DNA contamination. Total cDNA was synthesized as per the manufacturer’s instructions using the iScript cDNA synthesis kit (Bio-Rad). For expression analysis using real-time PCR, one-twentieth of the cDNA synthesis reaction was used as template per reaction. Tubulin expression was analyzed using primers Tubulin RT-F1 (5′-CTTCAAGTGCGGCATCAACTA-3′) and Tubulin RT-R2 (5′-TTAGTACTCCTCGACGTCCTC-3′). TR expression was analyzed using primers designed against LdBPK_050350.1 (TR-RT-F: 5′- CACAACATCAGCGGCAGCAAG-3′ and TR-RT-R: 5′-TCGGCGCTCGTCGGGTGGA-3′). Expression of TXN1 was analyzed using primers designed against LdBPK_291250.1 (TXN1-RT-F: 5′- GAGTTCTACGAGAAGCATCACA-3′ and TXN1-RT-R: 5′-TCAGCGTCGGAATCGATTCCA -3′). Expression of TXN2 was analyzed using primers designed against LdBPK_291240.1 (TXN2-RT-F: 5′- CAACAAACACGCGAAGTCGAAG-3′ and TXN2-RT-R: 5′-CGACGCCGATCAGCGTCGGA-3′). Expression of TXN3 was analyzed using primers designed against LdBPK_312000.1 (TXN3-RT-F: 5′- GACTACTACTGCCTGCCGTAC-3′ and TXN3-RT-R: 5′- GGCTGCTGCGGCTCTGCATC-3′). Expression of TxnPx was analyzed using primers designed against LdBPK_151140.1 (TxnPx-RT-F: 5′- GCCTACCGCGGTCTCTTCATC-3′ and TxnPx-RT-R: 5′-TTCCAGTTCGCGGGGCACAC-3′). Expression of genes in *set7*^−/−^ cells relative to in *set7*^+/+^ cells were determined using the 2^−△△Ct^ method (52), with tubulin gene expression serving as the internal control in each sample type. Expression of genes in H_2_O_2_-treated *versus* untreated cells were likewise determined using the 2^−△△Ct^ method using the tubulin gene as internal control. Real-time PCR experiments were done three times, with technical duplicates in each experiment. Values plotted are the average of three experiments and error bars show SD. Student’s *t* test was applied for analyzing statistical significance.

### Immunofluorescence analysis

Indirect immunofluorescence was carried out as described earlier (30). Briefly, exponentially growing promastigotes expressing LdSET7-FLAG were fixed with 2% paraformaldehyde, cell spreads prepared on poly-lysine coated coverslips, cells permeabilized with 0.1% Triton X-100, blocked with 5% chicken serum, incubated with anti-FLAG antibody for 2 h (Sigma Aldrich, 1:100 dilution), antibody washed off, incubated with Texas Red-labeled secondary antibody for an hour (Jackson Immunoresearch Laboratories, 1:100 dilution), and finally mounted in antifade solution containing 4′,6-diamidino-2-phenylindole (Vectashield, Vector Laboratories). Cells were viewed using the Leica TCS SP8X confocal microscope (with 100× (in oil) objective), and images captured and analyzed using the Leica Application Suite X (https://www.leica-microsystems.com/products/microscope-software/p/leica-las-x-ls/) software.

### TUNEL assay

To assess DNA strand breaks in *L. donovani* promastigotes (*set7*^+/+^ and *set7*^−/−^), cultures initiated at 1 × 10^6^ cells/ml from stationary phase cultures were grown to a cell density of 7 to 9 × 10^6^ cells/ml (day 3 in case of *set7*^+/+^ and day 6 in case of *set7*^−/−^ cells; *set7*^−/−^ cultures were initiated 3 days earlier to enable addition of H_2_O_2_ to all cultures at the same time), and H_2_O_2_ (100 μM or 200 μM) added. Cultures were incubated further for 5 h, and aliquots of 1 × 10^7^ cells removed immediately after to perform the TUNEL assay. For this, the cells were collected by centrifugation at 1448*g*, washed with 1× PBS, fixed with 2% paraformaldehyde, and cell spreads prepared on poly-lysine coated coverslips (2–4 × 10^6^ cells per coverslip). After cell permeabilization with 1× PBS-0.2% Triton X-100 for 10 min, the TUNEL assay was performed using the DeadEnd Fluorometric TUNEL System (Promega) as per the manufacturer’s instructions. Briefly, the adhered cells were incubated with the dUTP tailing reaction mix at 37 °C for an hour before stopping the reaction, washing the coverslips with 1× PBS to remove unincorporated dUTP, and mounting in antifade solution carrying 4′,6-diamidino-2-phenylindole. Cells were viewed using the Leica TCS SP8X confocal microscope (with 100× (in oil) objective), and images captured and analyzed using the Leica Application Suite X software.

### Measurement of ROS

ROS in *L. donovani* promastigotes were assessed using the DCFDA assay as described (24). Toward this, *L. donovani* promastigote cultures (*set7*^+/+^ and *set7*^−/−^) were initiated at 1 × 10^6^ cells/ml from stationary phase cultures, and H_2_O_2_ (100 μM) added when cells reached a density of 7 to 9 × 10^6^ cells/ml (day 3 in case of *set7*^+/+^ and day 6 in case of *set7*^−/−^; *set7*^−/−^ cultures were initiated 3 days earlier to enable addition of H_2_O_2_ to all cultures at the same time). Cultures were incubated further at 26 °C for 45 min, the medium was replaced with fresh H_2_O_2_-free medium and incubation at 26 °C continued. Aliquots of 1 × 10^7^ cells were removed at various time intervals thereafter to perform the DCFDA assay.

For this, the cells were collected by centrifugation at 1448*g*, medium was completely aspirated, and collected cells were washed twice in Hepes/NaCl buffer (21 mM Hepes (pH 7), 137 mM NaCl, 5 mM KCl, 6 mM glucose, 0.7 mM NaH_2_PO_4_) before suspending the cells in 1 ml of the same buffer. The DCFDA reagent (5 μM; Sigma Aldrich) was added to the cell suspension, reaction mixed by inverting the tube, and incubated in the dark at 26 °C for 45 min. This was followed by collecting the cells by centrifugation, washing them in the Hepes/NaCl buffer, and making a cell suspension in 1 ml of the buffer. Two hundred microliters aliquots of the cell suspensions were excited at 488 nm and fluorescence emission detected at 529 nm using a Tecan plate reader (Infinite 200 PRO).

Control reactions carried out without addition of DCFDA yielded no fluorescence. Control reactions carried out without cells gave “background” fluorescence readings, and the values of these control reactions (which were set up at every time point) were subtracted from the values obtained in the reactions with the two cell types (*set7*^*+/+*^ and *set7*^*−/−*^). At every time point, reactions with the two cell types were analyzed in case of both untreated and treated cells. The ratio between values obtained (after subtracting control reaction value) under treated *versus* untreated conditions was used as a measure of ROS activation in response to H_2_O_2_, for each cell type. The experiments were done thrice and mean values are presented in the bar charts; error bars indicate SD. Student’s *t* test was used to analyze statistical significance.

### Measurement of peroxidase activity

To analyze peroxidase activity in *L. donovani set7*^+/+^ and *set7*^−/−^ promastigotes, cultures were initiated at 1 × 10^6^ cells/ml from stationary phase cultures, and incubated to a cell density of 7 to 9 × 10^6^ cells/ml (*set7*^−/−^ cultures were initiated 3 days earlier so that it reached the cell density at the same time as *set7*^+/+^ cells), before adding H_2_O_2_ (100 μM) and allowing incubation at 26 °C for 5 h, then replacing the medium with fresh H_2_O_2_-free M199 and continuing incubation at 26 °C. Aliquots of 1×10^7^ cells were removed at various time intervals thereafter to perform the Amplex Red assay (53, 54). For this, the cell aliquots were washed with 1× PBS and resuspended in 500 μl assay mix (1× PBS carrying 64 μM digitonin, 10 μM Amplex Red (Invitrogen), 1 mM H_2_O_2,_ 1 mM protease inhibitors mix). The reaction mixes were incubated in the dark at 26 °C for 30 min, cell remnants removed by centrifugation, and fluorescence of the supernatant (200 μl aliquot) measured (excitation at 535 nm, emission at 590 nm).

Reactions carried out without addition of Amplex Red yielded no fluorescence. Reactions carried out without cells gave “background” fluorescence readings, and the values of these control reactions (set up with every time point) were deducted from the values obtained in the reactions with the two cell types (*set7*^*+/+*^ and *set7*^*−/−*^). At every time point, reactions with the two cell types were analyzed in case of both untreated and treated cells. The ratio between values obtained (after subtracting control reaction value) under treated *versus* untreated conditions was used as a measure of activation of peroxidase activity in response to H_2_O_2,_ for each cell type. The ratio between values obtained in *set7*-nulls *versus set7*^*+/+*^ in cells that had not been treated with H_2_O_2_ was used as a measure of basal peroxidase activity in *set7*-nulls relative to *set7*^*+/+*^ cells. The experiments were done thrice and mean values are presented in the bar charts. Error bars indicate SD, and student’s *t* test was used to analyze statistical significance.

### Macrophage infection experiment

Metacyclic parasites were incubated with macrophages (J774A.1) for infection as described previously (28, 29). Each experiment was carried out with three biological replicates and the data presented in the bar charts show the mean of three experiments, with error bars representing SD. The two-tailed student *t* test was applied to analyze statistical significance of the obtained data, and *p* values are mentioned in Figure legends.

## Data availability

The authors declare that all data supporting the findings of this study are available within the paper and its Supporting information files. Ld1S *set7* sequence has been deposited in GenBank. Accession no: OR479702. All raw data are available from the corresponding author upon request.

## Supporting information

This article contains supporting information.

## Conflict of interest

The authors declare that they have no conflicts of interest with the contents of this article.
