# Supplementary material for: The SET7 protein of Leishmania donovani moderates the parasite’s response to a hostile oxidative environment
Source: J Biol Chem. 2024 Feb 2;300(3):105720. doi: 10.1016/j.jbc.2024.105720 (PMC10907163; doi:10.1016/j.jbc.2024.105720)
Supplement: Supporting Figures S1–S7 and Table S1 [file mmc1.pdf]

The SET7 protein of *Leishmania donovani* moderates the parasite's response to a hostile oxidative environment

Jyoti Pal#, Varshni Sharma#, Arushi Khanna and Swati Saha*

Department of Microbiology

University of Delhi South Campus

New Delhi-110021

INDIA

# These two authors contributed equally to the work

****To whom correspondence may be addressed***

Running title: Role of *Leishmania donovani* SET7

Keywords: *Leishmania donovani*, trypanosome, SET domain, SET proteins, LdSET7, oxidative stress, protozoan parasite

Supporting Information:

Figure S1

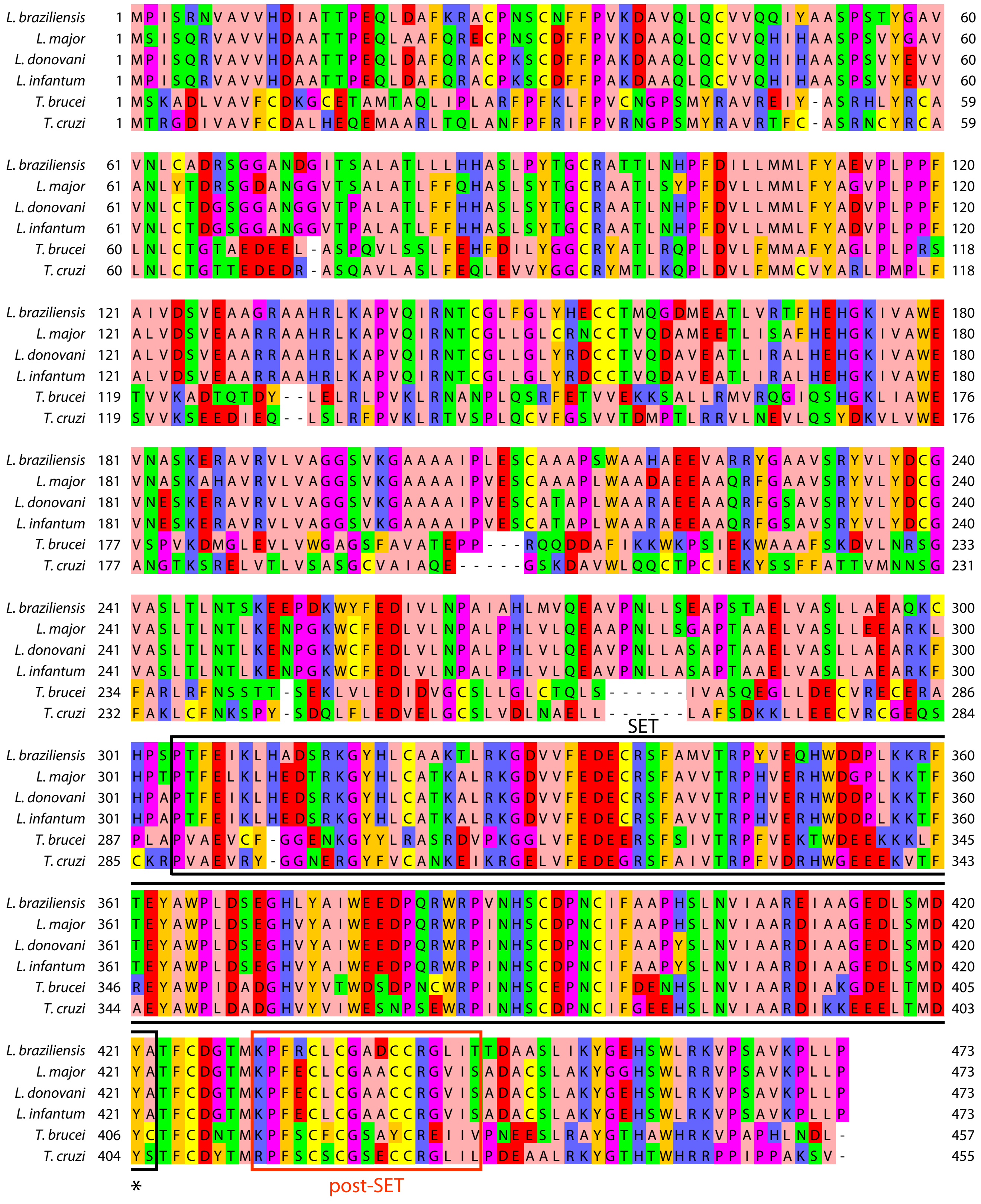

Figure S1. Analyis of LdSET7 amino acid sequence in comparison with amino acid sequences of SET7 of other trypanosomatid species. Clustal Omega analysis viewed using Jalview multiple alignment editor. SET and post-SET domains are demarcated with black and red boxes respectively. Colors indicate the physico-chemical properties of the amino acids. Pink- hydrophobic/aliphatic; red- acidic; purple- glycine / proline; yellow- cysteine; orange/ochre- aromatic; dark blue- basic; green- hydrophilic. Asterisk marks the conserved tyrosine residue involved in catalysis (Y421).

Figure S2

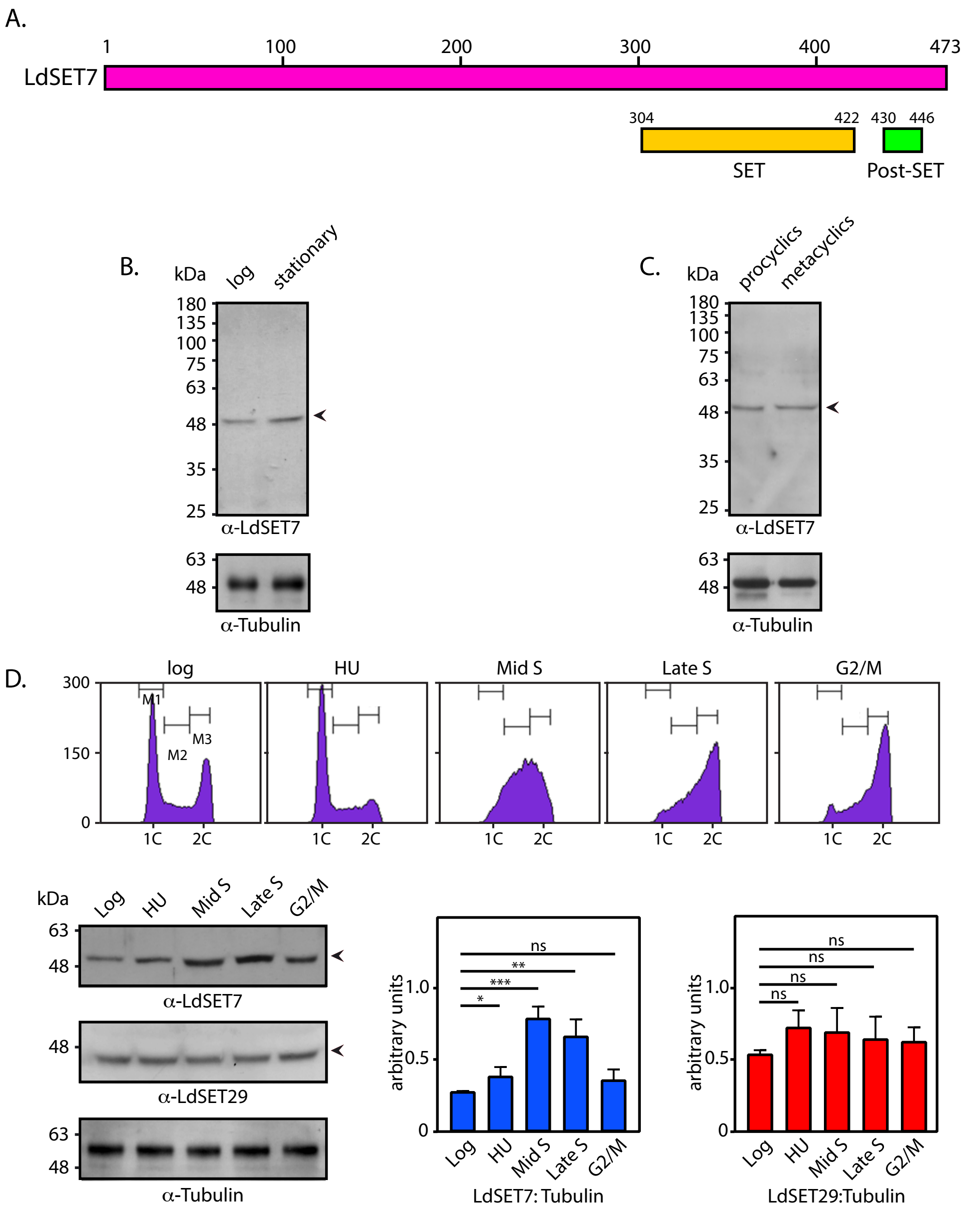

Figure S2. Analysis of expression of LdSET7 in *L. donovani* parasites. A. Schematic representation of the domains carried by LdSET7. B. Western blot analysis of whole cell lysates isolated from 4x10^7 logarithmically growing and stationary phase promastigotes using anti-SET7 antibodies (already available in the lab; 1:2500 dilution). Loading control: tubulin. C. Western blot analysis of whole cell lysates isolated from 4x10^7 procyclics and metacyclics using anti-SET7 antibodies. Loading control: tubulin. D. Western blot analysis of whole cell lysates isolated from synchronized *L. donovani* promastigotes (7x10^7 cells per time-point). Upper panels: Flow cytometry profiles depicting the different cell cycle stages at which extracts were isolated. Lower left panels: western blots using anti-SET7 and antibodies against LdSET29 (already available in the lab; 1:2500 dilution). LdSET7 was maximally expressed in S phase while LdSET29 was equivalently expressed at all stages. Loading control: tubulin. Lower middle and right panels: quantification of expression in western blots of three experiments, by Image J analysis. Average values are plotted, error bars depict standard deviation, two-tailed student's t-test was applied for statistical significance. *p value < 0.05, **p value < 0.005, ***p value < 0.0005, ns not significant.

Figure S3

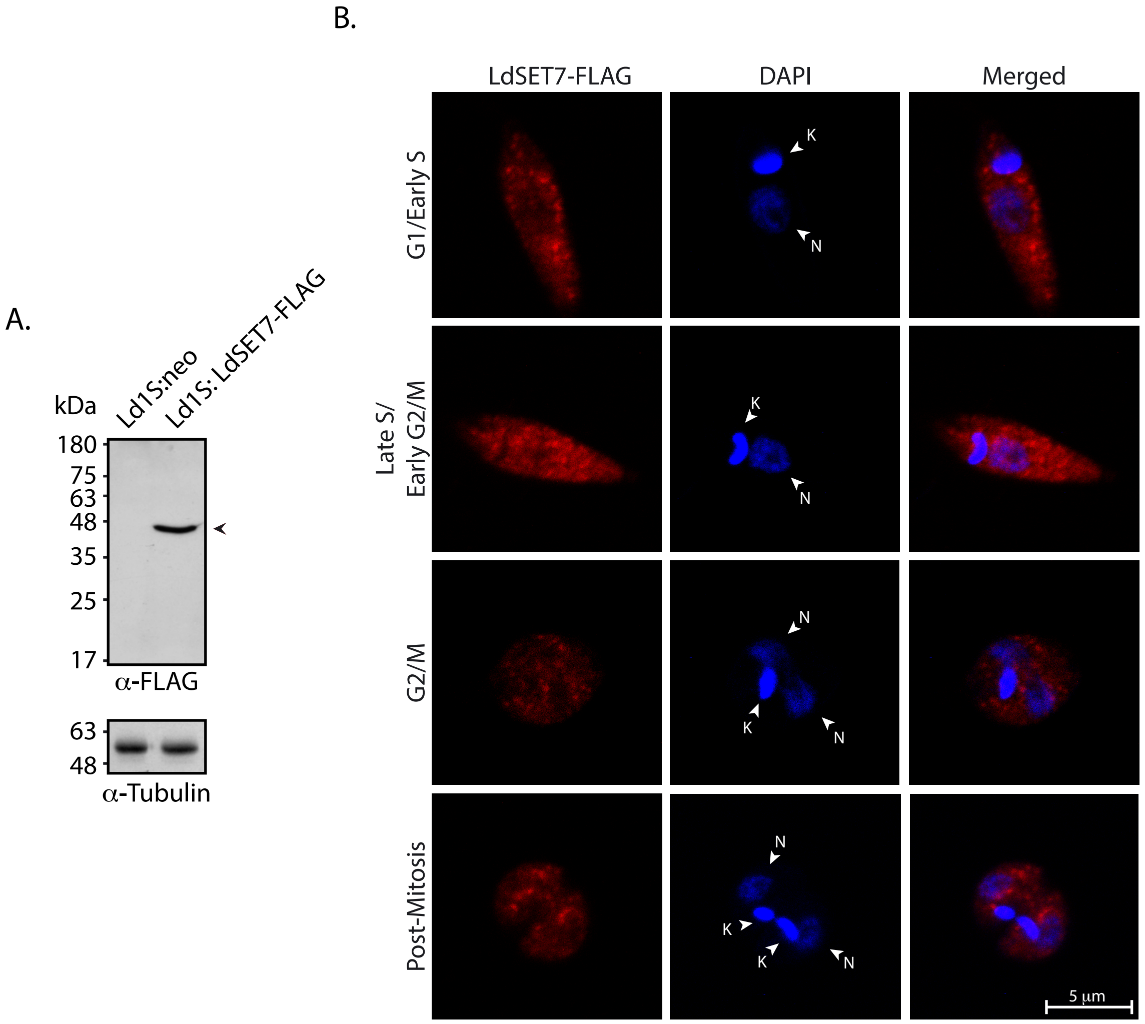

Figure S3. Analysis of subcellular localization of LdSET7. A. Western blot analysis of whole cell lysates isolated from Ld1S transfectant promastigotes, probed with anti-FLAG antibody (Sigma Aldrich, 1:1000 dilution). Loading control: tubulin. B. Immunofluorescence analysis of LdSET7-FLAG at different cell cycle stages using anti-FLAG antibody (1:100 dilution). N: nucleus. K: kinetoplast. Kinetoplast morphology and segregation pattern was used as cell cycle stage marker. One roundish/short kinetoplast, one nucleus (1N1K): G1/ early S phase. One elongated kinetoplast, one nucleus (1N1K): late S/ early G2M phase. Two nuclei, one kinetoplast (2N1K): G2M phase. Two nuclei, two kinetoplasts (2N2K): post-mitosis. Magnification bar: 5 μm.

Figure S4

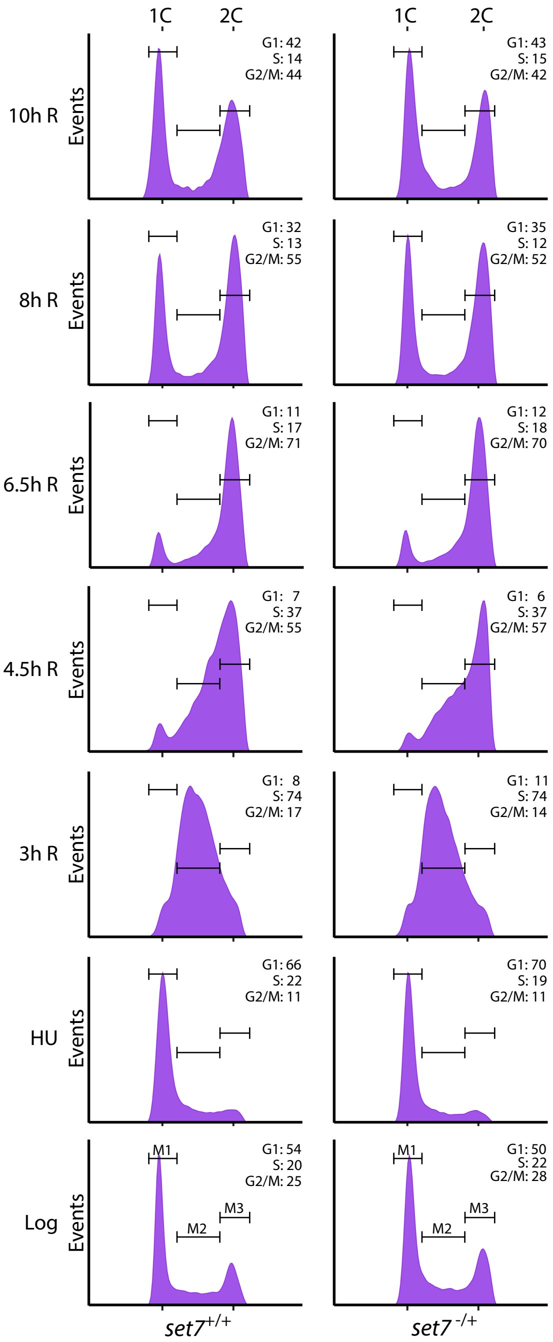

Figure S4. Flow cytometry analysis of HU-synchronized promastigotes. Time-points at which cells were sampled are indicated on the left of each row of histograms. R: release (3h R signifies 3 hours after release from HU-induced block). 30000 events were analyzed at every time-point. M1, M2 and M3 represent gating for cells in G1, S and G2/M respectively. Percent cells in each cell cycle phase are indicated in upper right-hand corner of each histogram. The experiment was done thrice, with comparable results, and data of one experiment is shown. *set7*$^{+/+}$ cells: Ld1S::hyg cells. *set7*$^{-/+}$ cells: *set7*-heterozygous knockout cells.

Figure S5

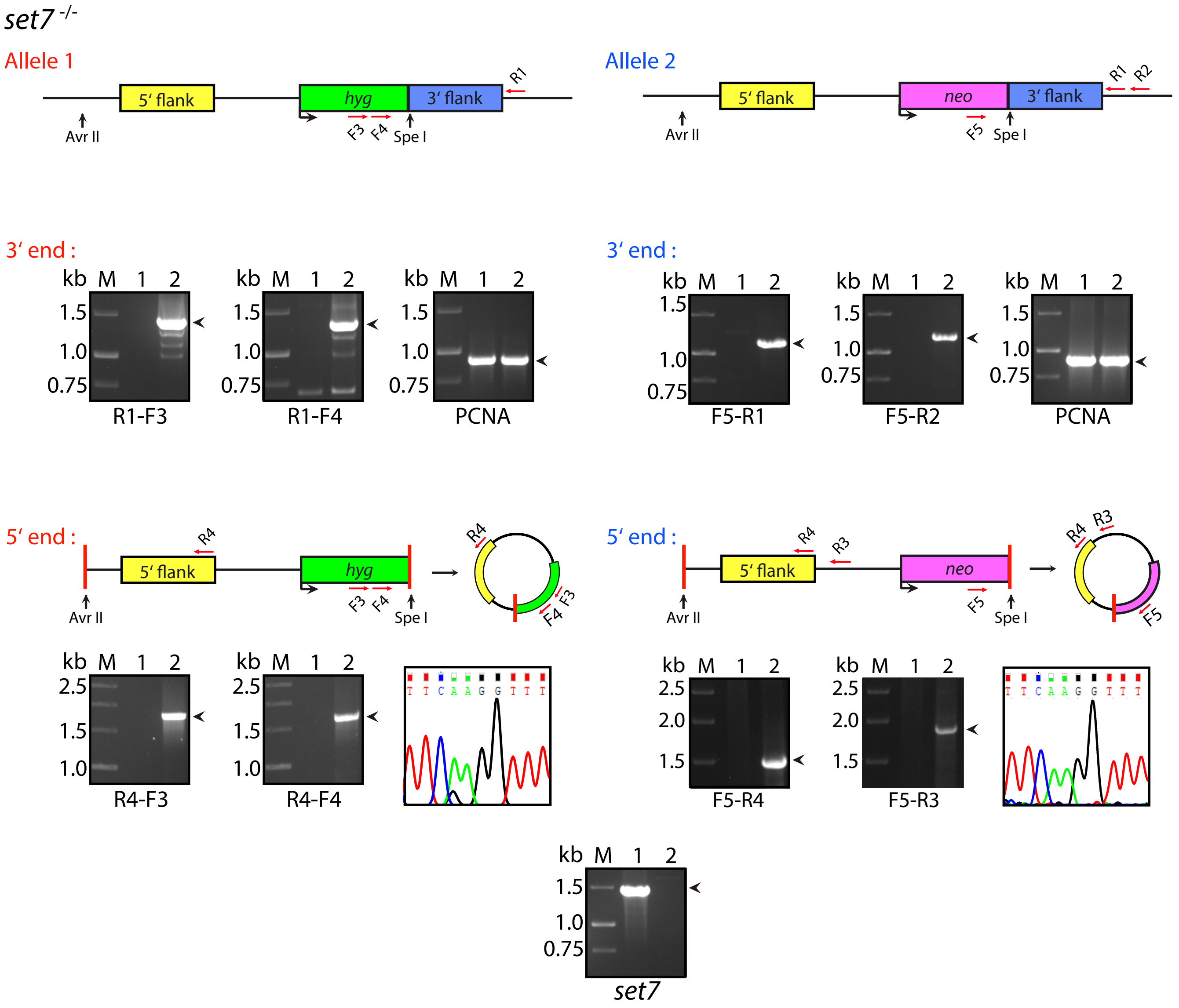

Figure S5. Creation of *set7 -/-*. Recombination at both ends was checked by PCRs (3′ends) / inverse PCRs (5′ends) across the deletion junctions. For inverse PCR analyses, genomic DNA was digested with AvrII and SpeI enzymes (positions marked on the line diagram) and self-ligated. Positions of primers used are indicated with arrows. F3, F4, F5: forward primers. R1, R2, R3, R4: reverse primers. PCNA served as input template DNA control. *set7*: PCR for *set7* gene. Lanes 1: Ld1S genomic DNA template. Lanes 2: *set7 -/-* genomic DNA template. M: DNA ladder.

Figure S6

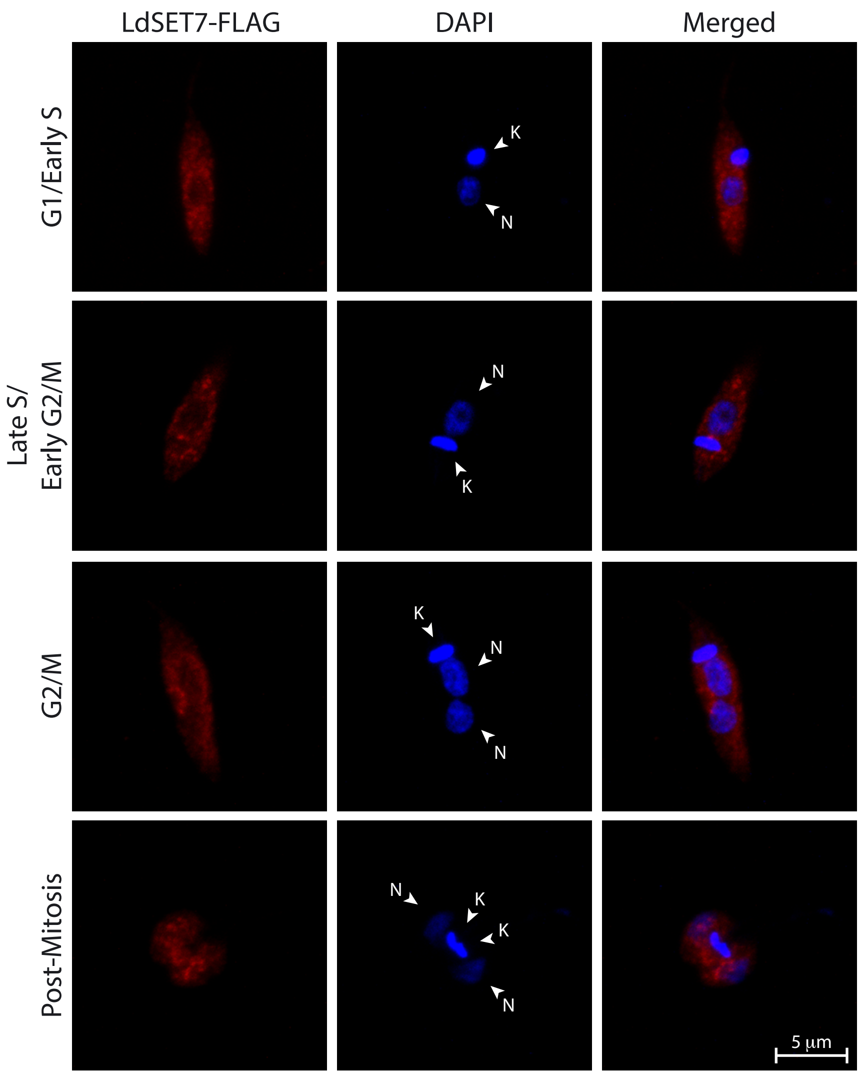

Figure S6. Effect of H_2O_2 on subcellular localization of SET7-FLAG. *set7*$^{+/+}$ cultures were initiated at 1x10^6 cells / ml, and hydrogen peroxide (100μM) added on reaching a cell density of ~ 7-9 x10^6 cells / ml (Day 3). Subcellular localization was analyzed after 5h exposure to H_2O_2. Magnification bar: 5 μm.

Figure S7

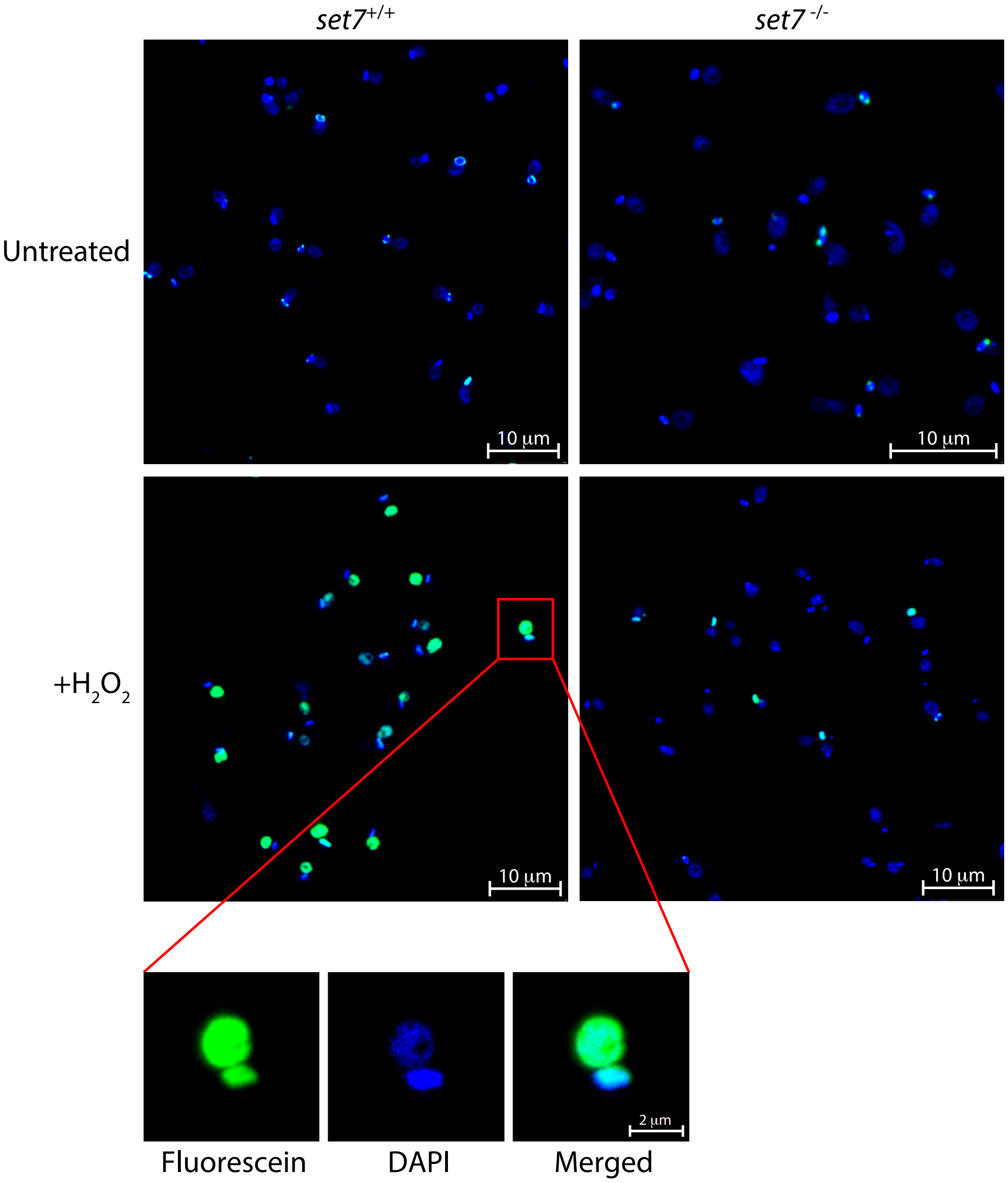

Figure S7: Analysis of DNA damage in *set7*$^{+/+}$ and *set7*$^{-/-}$ cells. Parasites were treated with 200 µM H_2O_2 for 5 hours and analyzed by TUNEL reaction. Each panel depicts merged image of fluorescein-UMP labelled DNA and DAPI fluorescence. Magnification bar: 10 µm. Inset shows magnification of a single cell. Green fluorescence: fluorescein-labelled dUMP, blue fluorescence: DAPI. Magnification bar: 2 µm.

Table S1: Analysis of parasites by TUNEL

	% cells with labeled nuclei	
**Treatment**	**Parasite type**	
	Set7$^{+/+}$	***Set7*** $^{-/-}$
Untreated	2.5 ± 0.5	4.5 ± 0.5
100 µM H_2O_2	13.3	4
200 µM H_2O_2	95.5 ± 0.5	3 ± 1

Table S1: Analysis of parasites by TUNEL assay. Percent cells with labelled nuclei were scored. The data from two independent experiments have been tabulated. Percent values are average of two experiments.
